# Synergistic Combinations of Native Australian Plants For Skin Inflammation and Wound Healing

**DOI:** 10.3390/biomedicines13071754

**Published:** 2025-07-17

**Authors:** Rotina Kapini, Dennis Chang, Gerald Münch, Lisa Carroll, Xian Zhou

**Affiliations:** 1 NICM Health Research Institute, Western Sydney University, Westmead, NSW 2145, Australia; 30067405@westernsydyney.edu.au (R.K.); d.chang@westernsydney.edu.au (D.C.); 2 School of Medicine, Western Sydney University, Campbelltown, NSW 2560, Australia; g.muench@westernsydney.edu.au; 3 NATIVE EXTRACTS Pty Ltd., Alstonville, NSW 2477, Australia; lisa@nativeextracts.com

**Keywords:** Australian native plants, skin inflammation, oxidative stress, wound healing, synergy, combination therapy

## Abstract

**Background:** Inflammation and oxidative stress are key mechanisms in underlying skin conditions like psoriasis and eczema. While many plants, including Australian native plants, are proposed to target these pathways due to their phytochemical content, studies on whole extracts and their synergistic effects remain limited. **Objectives**: This study aimed to investigate individual and combined effects of whole plant extracts on skin protection and healing, focusing on their anti-inflammatory and antioxidant properties. **Methods**: The antioxidant potential of the individual and combined plant extracts were investigated on 2,2-diphenyl-1-picrylhydrazyl (DPPH) and reactive oxygen species (ROS) assay followed by luciferase assay in MCF-7 AREc32 cells for nuclear factor erythroid 2-related factor 2 (Nrf2) activation. The anti-inflammatory activities were investigated on lipopolysaccharide (LPS)-induced RAW 264.7 murine macrophages for the inhibition of nitric oxide (NO), tumour necrosis factor (TNF)-α, and interleukin (IL)-6. Synergistic interaction was determined by the combination index model (CI < 1). Combination(s) showing synergistic and optimal activity were further investigated on LPS-induced human dermal fibroblasts (HDF) cells for IL-6 inhibition and wound healing activity. **Results**: Three of the tested Australian native plant extracts demonstrated prominent antioxidant and anti-inflammatory activities including bitter orange, mountain pepper berry and native river mint. In particular, their three-way combination (1:1:1, *w*/*w*) showed prominent synergistic (CI < 1) in reducing NO and IL-6, along with enhanced Nrf2 activation. In LPS-inflamed HDF cells, the combination maintained synergistic inhibition of IL-6 levels and promoted wound healing response. **Conclusions**: These findings highlight the therapeutic potential of Australian native plant as a whole extract for skin protection and repair attributed to antioxidant and anti-inflammatory activities. The observed synergistic anti-inflammatory and antioxidant effects support their use in the development of new cosmetic formulations for skin.

## 1. Introduction

Oxidative stress and inflammation contribute to the pathogenesis of skin damage and diseases like eczema, psoriasis and lupus [1,2,3,4]. Oxidative stress occurs when the excessive production of reactive oxygen species (ROS) overwhelms the body’s antioxidant defence mechanisms [5,6]. ROS include mediators like superoxide (O_2_^−^), hydroxyl groups (OH), hydrogen peroxide (H_2_O_2_) and hypochlorous acid (HOCI) [7]. Studies suggest that elevated levels of ROS can hinder cell growth processes in wounds, leading to further tissue damage and delayed wound healing processes [8]. Research also demonstrates the interaction between ROS and inflammation in skin damage via ROS stimulated-nuclear factor kappa B (NF-κB), mitogen-activated protein kinase (MAPK) and the Janus kinase-signal transducers and activators of transcription (JAK-STAT) pathways [9,10,11]. These pathways, ultimately, produce pro-inflammatory mediators like nitric oxide (NO), interleukin-6 (IL-6) and tumour necrosis factor-α (TNF-α) [12]. Presently, conventional interventions for managing oxidative stress and inflammation in skin conditions include sunscreen [13], topical corticosteroids [14] and non-steroidal anti-inflammatory drugs [15]. However, these interventions show limited efficacy [16,17] and struggle to address the complex pathophysiology of skin diseases [18,19]. Furthermore, they are often associated with various adverse effects, which further limit their long-term use and overall effectiveness [20,21,22,23,24].

Combination therapy via a synergistic approach offers promising therapeutic outcomes by simultaneously targeting inflammation and oxidative stress in a multi-target mechanism [25]. Synergy, defined as the interaction between two or more agents, that produces a greater therapeutic effect than the sum of their individual effects [25], can manifest through enhanced potency, reduced toxicity and combined multi-target action [26]. Researchers can rigorously evaluate synergism using mathematical models like the combination index (CI).

Botanical species, both native to Australia and other regions, present significant potential for developing novel dermatological products due to their abundance of bioactive compounds and multi-target bioactivities [27,28]. A prior literature survey identified nine such plants—some endemic Australian flora and some non-Australian species—that exhibited potent anti-inflammatory and antioxidant properties due to their bioactive compounds.

Aniseed myrtle (*Backhoisia anisata* or *Syzygium anisatum*) contains high phenolic content (55.9 mg gallic acid equivalents/g dry weight) which contributes to its strong potent antioxidant activity such as 2,2-diphenyl-1-picrylhydrazyl (DPPH) scavenging activity and oxygen free radicals [29,30]. Guo et al. also reported that Aniseed myrtle exerts significant anti-inflammatory effect by reducing inducible nitric oxide (iNOS) and cyclooxygenase (COX) in lipopolysaccharide (LPS)-stimulated RAW 264.7 murine macrophage cells at non-toxic concentrations [31].

Bitter orange (*Citrus aurantium*) is known to contain potent compounds such as linalyl acetate which decreased elevated serum levels of TNF-α, interleukin (IL)-1β and prostaglandin E2 and increased glutathione levels in the carrageenan-induced rat paw oedema model at 100 mg/kg and 200 mg/kg [32]. Other orange flavonoids, notably nobiletin, naringin and hesperidin, have also inhibited pro-inflammatory cytokines at tested concentrations from 6.25 µg/mL to 50 µg/mL in LPS-stimulated RAW 264.7 cells [33]. This anti-inflammatory effect was induced by inhibiting MAPK/NF-κB signalling pathways and mRNA expressions of COX-2 and iNOS [33].

Blue butterfly pea (*Clitoria ternatea*) is another plant with potent antioxidant activity. A study extracted 4.0 mg of anthocyanins from its flower which displayed prominent DPPH radical scavenging effects and 75–80% inhibition of ROS in RAW 264.7 cells [34]. Another flower water extract of blue butterfly pea was able to reduce cytotoxicity effects in hydrogen peroxide (H_2_O_2_)-nduced HaCaT cells and significantly (*p* < 0.05) reduced DNA damage in UV-exposed HaCaT cells at 100, 250 and 500 µg/mL [35]. A review noted that the petal extracts ameliorated LPS-induced RAW 264.7 cells via the inhibition of COX-2 levels and NF-κB translocation [36].

Blue cypress (*Callitris columellaris* or *Callitris intratropica*) is another native plant of Australia which possesses high DPPH scavenging activity (55–90%) in its leaf extract [37]. It is twice as potent as synthetic ascorbic acid [37]. Essential oils derived from blue cypress leaves have also shown significant reductions of oedema paw volume in mice at 25 mg/kg [38].

Emu bush (*Eremophila longifolia*) is also a traditionally used Australian plant species for antiseptic and antibacterial therapy for wound and skin infections [39]. In a more detailed investigation by Sadgrove, 1 mg of partially pyrolysed oils of emu bush showed 30–700 times greater radical scavenging activity than non-pyrolysis oils [40].

Mountain pepper berry (*Tasmannia lanceolata*), another native plant to Australia, offers high antioxidant content. For instance, the anthocyanin levels in the plant were four times greater than blueberries [41]. It also contains anti-inflammatory flavonoids like quercetin and phenolic compounds such as chlorogenic acid, which possess potent skin-protective properties [22].

River mint (*Mentha australis*) is traditionally used for coughs and colds by the Australian Indigenous people [42]. Despite the lack of direct evidence in anti-inflammatory and antioxidant activities, Tang, Konczak and Zhao have identified that the plant contains high antioxidant potential by demonstrating high DPPH free radical scavenging effects and showing high phenolic contents like naringin, kaempferol and neoericitrin [42].

Pineapples (*Ananas sativa* or *Ananas comosus*), although not native but are grown in subtropical regions of Australia, contain sources of antioxidants, vitamins, organic acids and minerals [43]. A study by Hoosain and Rahman found that methanolic extracts of pineapple displayed effective elimination of free radicals for DPPH, with an inhibitory activity of >20% [44].

Lastly, Kakadu plum (*Terminalia ferdinandiana*) is a well-known Australian plant due to its significant vitamin C content [45]. An in vitro investigation on the kakadu plum purified phenolic content (100 µg/mL, 200 µg/mL and 400 µg/mL) dose-dependently and reduced nitrate and prostaglandin E2 in LPS-induced RAW 264.7 cells, followed by providing significant protective effects from H_2_O_2_-induced apoptosis [46]. Kakadu plum also demonstrated strong dose-dependent antioxidant potential at 100 µg/mL, 200 µg/mL and 400 µg/mL by exhibiting high free radical scavenging activity and activation of the Nrf2/Keap1 pathway [46].

Although these plants contain potential sources of vital anti-inflammatory and antioxidant properties due to their active compounds, there is still limited evidence on the direct investigations of these plants as whole plant extracts. Moreover, no existing research in the literature has examined the possible synergistic combinations of these Australian native plants to combat oxidative stress and inflammation for skin-related conditions.

This study aims to examine the anti-inflammatory and antioxidant effects of the nine listed Australian plants and further develop synergistic combinations of these plants for skin protection and healing ability.

## 2. Materials and Methods

**Figure 1:**
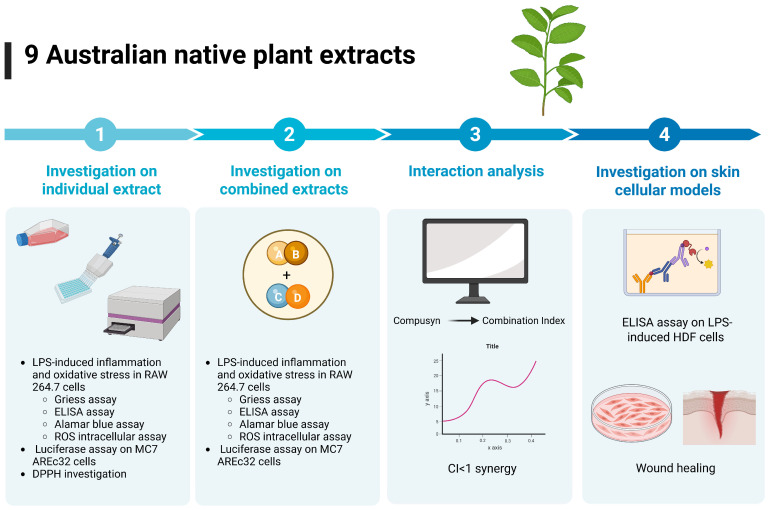
Overview of research design and methodology. The NPEs were evaluated for anti-inflammatory and antioxidant activity in LPS-induced RAW 264.7 cells using the Griess assay, ELISA, ROS intracellular assay. Cell viability was determined by Alamar blue assay. The NPEs were also subjected to luciferase assay for Nrf2 activation and DPPH investigation. Based on individual efficacy, the NPEs were combined in 1:1 or 1:1:1 by weight ratio and re-tested in the same cellular models. Interactions between the extracs were analysed by Compusyn 2.0 and the combinations with most synergistic effects were further evaluated in skin cell models for anti-inflammatory activity and wound healing ability. Figure was generated using BioRender template.

An overview of the research design and methodology is presented in Figure 1.

### 2.1. Preparation of Australian Native Plant Extracts

In this study, nine plant extracts (NPEs), commercially known as cellular extract concentrate variants, were supplied by NATIVE EXTRACTS Pty Ltd, Alstonville, NSW, Australia. These included extracts from aniseed myrtle (*Backhoisia anisata* or *Syzygium anisatum*), bitter orange (*Citrus aurantium*), blue butterfly pea (*Clitoria ternatea*), blue cypress leaf (*Callitris columellaris* or *Callitris intratropica*), emu bush (*Eremophila longifolia*), mountain pepper berry (*Tasmannia lanceolata*), native river mint (*Mentha australis*), pineapple (*Ananas sativa* or *Ananas comosus*) and kakadu plum (*Terminalia ferdinandiana*). The plants were extracted as a glycerine extract in a viscous solution. The phytochemical constituents for the NPEs, as listed in Table 1, were obtained from the product information provided by NATIVE EXTRACTS Pty Ltd. The estimated starting concentrations of the NPEs were calculated by dividing the net weight by the weight of 1 mL. The starting concentrations for these extracts were as follows: 1.25 g/mL, 1.18 mg/mL, 1.18 g/mL, 1.20 g/mL, 1.21 g/mL, 1.18 g/mL, 1.23 g/mL, 1.32 g/mL and 1.17 g/mL. Based on individual potency, bitter orange, mountain pepper berry and native river mint were selected to combine in two-way or three-way combinations at either a 1:1 or 1:1:1 by weight ratio at a starting concentration of 1.18 g/mL and sequentially diluted by a half. The detailed information for the nine NPEs can be found in Supplementary Material Table S1.

### 2.2. Cell Culture

Murine RAW 264.7 macrophages and MCF-7 AREc32 were kindly provided by Prof. Gerald Münch in the School of Medicine at Western Sydney University [47,48]. Human dermal fibroblasts (HDF) were kindly provided by Dr. Katie Dixon in the School of Medical Sciences, University of Sydney [49]. Cell maintenance and culture followed the method from our previous study [34]. The cells were grown in T75 cm^2^ culture flasks (Interpath Services, Sydney, Australia) in Dulbecco’s Modified Eagle Medium (Thermo Fisher Scientific, North Ryde, Australia) containing foetal bovine serum (10%) (FBS; CellSera, Rutherford, Australia) and pen-strep antibiotics (2%) (Sigma-Aldrich, Sydney, Australia). Cells were incubated in humidified conditions with 5% CO_2_ with the DMEM being replaced every 3–4 days. Cultured cells were passaged with 90% confluency and were suspended from flasks using 0.25% trypsin (Thermo Fisher Scientific, North Ryde, Australia) when performing experiments.

### 2.3. Detection of Nitrite by Griess Assay

The NO production in RAW 264.7 cells was measured using the Griess assay using a method from a previous study [47]. LPS from *Escherichia coli* and O55:B5 purified by phenolic extraction (Sigma-Aldrich, Kingsgrove, Australia) were used to induce NO production. RAW 264.7 cells (1 × 10^6^/mL) were briefly seeded in a 96-cell culture plate (Starstedt, Kingsgrove, Australia) and incubated for 24 h before treating with individual or combined NPE followed by a 2 h incubation. The cells were further stimulated with LPS (50 ng/mL) and co-incubated for another 18 h. After stimulation, the cell supernatants (50 µL) were collected and mixed with equal volumes of Griess reagents containing 1% sulphanilamide in 5% phosphoric acid and 0.1% N-(1-naphthyl) ethylenediamine dihydrochloride in Milli-Q water (BaneBio LLC, Frederick, MD, USA). NO was measured at 540 nm absorbance using a CLARIOstar microplate reader (BMG Labtech, Mornington, Australia).

### 2.4. Detection of TNF-α and IL-6 Expressions by ELISA Assay

The cell supernatants from LPS-induced RAW 264.7 and HDF were determined for TNF-α and IL-6 using the commercial sandwich ELISA kits (Biolegend TNF-α Mouse, #430916; IL-6 Mouse, #431315; IL-6 Human, #430504, San Diego, CA, USA) based on previous study [47]. An assay was performed following the manufacturer’s protocol. TNF-α and IL-6 internal standards were diluted in the diluent buffer to create a standard curve. The biotinylated second antibody, avidin peroxidase conjugate and 3,3′,5,5′-tetramethylbenzidine (TMB) were utilised to measure the levels of TNF-α and IL-6. After 15–20 min, 0.5 M sulphuric acid stopped the reaction, and a subsequent measurement was carried out using a CLARIOstar microplate reader at 450 nm and 570 nm. The 570 nm readings were subtracted from 450 nm readings to eliminate background noise.

### 2.5. 2,2-Diphenyl-1-picrylhydrakzl (DPPH) Assay

The DPPH cation method was used to evaluate the radical-scavenging effect of the NPEs [50]. The donating ability of the hydrogen atom in the samples was determined by the decolourisation of the methanol solution of the DPPH, which produces a purple colour that fades to a yellow colour [50]. This reaction shows the presence of antioxidants. DPPH solution in methanol was prepared and added to the extracts at different concentrations. The DPPH mixture was vortexed and left at room temperature in the dark for 30 min. The mixture was read for absorbance spectrophotometrically at 517 nm.

### 2.6. Detection of Intracellular ROS Levels

ROS levels were detected using an ROS-sensitive fluorescence indicator known as 2,7 -dichlorofluorescein diacetate (DCF-DA) on LPS-induced RAW 264.7 cells [51]. Following the overnight activation of LPS, the supernatants were removed, and the plate was washed with autoclaved phosphate buffered saline (PBS). DCF-DA (10 µM) (Sigma-Aldrich, Australia) was added to the cells and incubated in the dark for 30 min at a temperature of 37 °C. DCF-DA was rinsed with PBS and the plate was measured for fluorescence using the CLARIOstar microplate reader at 485 nm excitation and 535 nm emission.

### 2.7. Detection of Nrf2 Activation by Luciferase Assay

The Nrf2 luciferase assay was conducted following our previous study [48]. MCF-7 *ARE*c32 cells were used for this assay because they contain a stably integrated *antioxidant response element* (*ARE*)-driven luciferase reporter gene [48]. Upon activation and nuclear translocation, Nrf2 binds to the *ARE*, inducing luciferase expression, which produces a quantifiable luminescent signal. The MCF7-*ARE*c32 cells were seeded at 1 × 10^6^ cells/mL in a 96-well plate and incubated for 24 h. The cells were treated with individual or combined NPE and sequentially diluted two-fold in the 96-well plate and incubated for another 24 h. The NPEs were investigated against a control group (cells with 0.1% DMSO in media) and positive control, tert-butylhydroquinone (TBHQ, Sigma-Aldrich, Australia), a known Nrf2 activator [52]. After incubation, the supernatants were mixed with 20 µL of triton lysis buffer (TLB) containing 0.04 M Tris-Base, 0.1 M Tris-HCl, 5 M NaCl and 1 M MgCl_2_, shaken for 10 min and placed in −20 °C for 20 min. The cells were transferred to a 96-well white plate and 100 µL of luciferase buffer containing TLB, 80 mg/mL of D-luciferin, 10 mM of ATP, 1 M of DTT and 10 mM of Coenzyme A was added to the cells. The bioluminescence was read at emission 555–70 nm in the CLARIOstar reader to quantify the Nrf2 activation.

### 2.8. Cell Viability and Cytotoxicity Test via Alamar Blue Assay

A cell viability assay was conducted to examine the safe concentrations of individuals and combined NPEs on cell survival. For the Alamar blue assay, the method from our previous study was followed [47]. Alamar blue solution containing 10% resazurin (Sigma-Aldrich, Australia) in DMEM serum-free media was added to the cells, and the plate was incubated for 2 h at 37 °C. The fluorescence was measured at 530 nm excitation and 590 nm emission using the CLARIOstar microplate reader.

### 2.9. Wound Healing Assay

A wound healing assay was conducted following the method from Tan et al. [53]. HDF cells were seeded (2 × 10^5^ cells/mL) in a 24-well plate and incubated until cells formed a monolayer. The cell monolayer in each well was wounded in straight cross-shaped lines using a sterile 200 µL pipette tip and ruler as a guide. The intersection of the wound was marked on the underside of the well with a marker to view under the microscope. Cell debris was washed with autoclaved PBS. The plate was viewed under an EVOS M5000 Cell Imaging Systems (Thermo Fisher Scientific, North Ryde, Australia) at ×4 magnification, and an image of the wound was captured and recorded at 0 h. The NPEs were added to the wounded cells before incubating for 24 h. As the control, wounded monolayers were incubated with serum-free DMEM. Following incubation, the cell media were removed, and the wound was viewed again under the EVOS system at ×4 magnification. An image of the wound was captured and recorded as 24 h. The wound images were analysed using the ImageJ 1.54g software (National Institute of Health, Bethseda, MD, USA) with the Wound Healing Size Tool plugin to quantify the wound gap [54,55]. The software generated the wound gap area in pixels from images captured at 0 and 24 h. Wound closure was calculated as a percentage by subtracting the area at 24 h from the area at 0 h, dividing by the area at 0 h, and multiplying by 100.

### 2.10. Synergy Determination

The CI model was used to determine the interactions of the NPE combinations by CompuSyn software 2.0 (ComboSyn, Inc., Paramus, NJ, USA). The dose–response data from the in vitro investigations were entered into the CompuSyn software, and CI and Fa values were generated. These values were entered into GraphPad Prism 10.0 to create a CI-Fa curve. Fa values refer to the effect level based on the assay, and the CI values were used to determine the interaction, where CI < 1, CI = 1 and CI > 1, respectively, refer to synergistic, additive and antagonistic interaction [56].

### 2.11. Statistical Analysis

Data analysis was performed in MS Excel 2016 software and GraphPad Prism 10.0 to construct a dose–response curve for NO, TNF-α, IL-6 and ROS assays and obtain the IC_50_ and LC_50_ values. The therapeutic index for the NPE in each assay was calculated as dividing the LC_50_ by IC_50_ value, to evaluate their relative safety and efficacy. Results were expressed as mean ± standard error of the mean (SEM). The statistical differences among groups were assessed by the analysis of variance (ANOVA), where significance is determined when the *p*-value is less than 0.05.

## 3. Results

### 3.1. Anti-Inflammatory Effects of the Nine Individual NPE

The study assessed the anti-inflammatory properties of nine individual extracts by evaluating their inhibitory effects on NO, TNF-α and IL-6 expressions in LPS-induced RAW 264.7 cells. In the presence of LPS (50 ng/mL), the cells produced substantial amounts of nitrite (10.05 ± 1.01 µg/mL), TNF-α (46.02 ± 10.51 ng/mL) and IL-6 (159.76 ± 40.44 ng/mL) which were detected in the cell supernatants.

All nine NPEs inhibited the production of NO, TNF-α and IL-6 in a dose-dependent manner (See Supplementary Material Figures S1–S3). Among them, Aniseed myrtle (#1) demonstrated the most potent inhibitory effects across all inflammatory biomarkers, with IC_50_ values of 1.11 ± 0.40 mg/mL for NO, 4.38 ± 2.12 mg/mL for TNF-α and 3.42 ± 1.42 mg/mL for IL-6. However, it also showed the highest cytotoxicity in RAW 264.7 cells (LC_50_: 19.33 ± 3.24 mg/mL). Native river mint (#7) followed by mountain pepper berry (#6) and bitter orange (#2) also exhibited strong NO inhibition, with IC_50_ values of 3.13 ± 1.31 mg/mL, 4.44 ± 0.51 mg/mL and 7.44 ± 2.93 mg/mL, respectively. In addition, mountain pepper berry (#6) and bitter orange (#2) continued to display notable anti-inflammatory activity by inhibiting TNF-α (#6: IC_50_: 13.32 ± 3.22 mg/mL and #2: IC_50_: 15.70 ± 1.70 mg/mL) and IL-6 (#6: IC_50_: 13.49 ± 6.83 mg/mL and #2: IC_50_: 13.55 ± 4.02 mg/mL).

Overall, bitter orange (#2) achieved the highest therapeutic indices (NO: 28.17, TNF-α: 13.34 and IL-6: 15.47) across the inflammatory markers due to its high LC_50_ value (>100 mg/mL). Aniseed myrtle (#1) followed with indices 17.80 (NO), 4.50 (TNF-α) and 5.78 (IL-6), while mountain pepper berry (#6) and bitter orange (#2) showed moderate indices (#6:NO: 10.53, TNF-α: 3.51 and IL-6: 3.46; 7: NO: 18.12, TNF-α: 2.42 and IL-6: 1.77). Although pineapple (#8) presented the lowest cytotoxicity among all extracts, it also exhibited the weakest anti-inflammatory activity. Summarised results of anti-inflammatory activities are shown in Table 2.

### 3.2. Antioxidant Effects of the Nine Individual NPE

The study also evaluated the potential antioxidant activities of the nine NPEs using the DPPH assay to measure their free radical scavenging capacity (See Supplementary Material Figure S4). The study normalised the data by calculating the DPPH scavenging effect relative to a ‘blank’ control, which the study set at a 100% scavenging effect.

Only a select few of the NPEs displayed potent scavenging activity. Mountain pepper berry (#6) exhibited a prominent effect, with an IC_50_ value of 0.945 ± 0.06 mg/mL. Aniseed myrtle (#1) (IC_50_: 1.85 ± 0.71 mg/mL), bitter orange (#2) (IC_50_: 2.08 ± 0.34 mg/mL) and native river mint (#7) (IC_50_: 9.65 ± 0.37 mg/mL) also demonstrated notable DPPH radical scavenging activity. Extracts that were least potent were Emu bush (#5) and pineapple (#8), exhibited minimal activity, with an IC_50_ > 1000 mg/mL.

The study also conducted an ROS assay to determine the extract’s ability to reduce oxidative stress induced by LPS in RAW 264.7 cells. All NPEs inhibited ROS generation in a dose-dependent manner (See Supplementary Material Figure S5). Aniseed myrtle (#1) produced the strongest inhibition (IC_50:_ 3.21 ± 0.76 mg/mL) but also showed the highest cytotoxicity (LC_50_: 19.78 ± 4.25 mg/mL). This was followed by native river mint (#7) (IC_50_: 4.95 ± 1.50 mg/mL), mountain pepper berry (#6) (IC_50_: 6.56 ± 1.04 mg/mL) and bitter orange (#2) (IC_50_: 9.69 ± 2.82 mg/mL) (Table 3). Bitter orange (#2) achieved the highest therapeutic index of 21.63 for ROS inhibition. Native river mint (#7) and mountain pepper berry (#6) followed with therapeutic indices of 11.45 and 7.13, respectively. In contrast, pineapple (#8) along with blue butterfly pea (#3) yielded therapeutic indices below 1, indicating limited antioxidant effectiveness.

To explore potential mechanisms of action, the current study performed a luciferase assay to determine whether the extracts activated Nrf2, a key regulator of antioxidant responses. The nine NPEs generally demonstrated weak Nrf2 activation, with a 1-3-fold increase within safe concentrations (See Supplementary Material Figure S6). However, mountain pepper berry (#6), triggered the highest mean Nrf2 activation (3.47 ± 0.85-fold increase) at 36.87 mg/mL (*p* < 0.01 vs. blank). Native river mint (#7) and bitter orange (#2) followed, producing peak Nrf2 activation of 2.82 ± 0.60-fold at 38.43 mg/mL (*p* < 0.01 vs. blank) and 2.22 ± 0.79-fold at 36.87 mg/mL, respectively. Summarised results of antioxidant activities are shown in Table 3.

### 3.3. Synergistic Anti-Inflammatory Effects of NPE Combinations

The current study selected three individual NPEs to develop synergistic combinations based on their respective anti-inflammatory and antioxidant potencies. Bitter orange (#2) demonstrated the highest therapeutic indices in reducing NO, TNF-α and IL-6 levels. Mountain pepper berry (#6) earned selection based on its broad inhibitory effects on all three inflammatory mediators, strong DPPH radical scavenging activity and highest Nrf2 activation. Native river mint #7 was selected due to its second-highest therapeutic index in reducing NO and ROS production, along with its Nrf2 activation. These bioactivities suggested that the three extracts could exhibit synergistic and multi-target behaviour.

To investigate this, the study evaluated synergistic interactions in NO, TNF-α and IL-6 inhibition using pair-wised combinations of bitter orange (#2), mountain pepper berry (#6) and native river mint (#7) at a 1:1 by weight ratio, as well as triple combination (#2+6+7) in a 1:1:1 by weight ratio.

The four NPE combinations showed LC_50_ values above 50 mg/mL (Table 4). Among the combinations, the #6+7 combination exhibited the lowest LC_50_ value (58.96 ± 5.02 mg/mL), followed by #2+6 (LC_50_: 72. 85 ± 7.65 mg/mL) and #2+6+7 (LC_50_: 80.97 ± 3.76 mg/mL). The #2+7 combination produced the highest LC_50_ value of 91.81 ± 18.67 mg/mL. Compared to the individual extracts, #2+6+7 significantly reduced their individual cytotoxicity (*p* < 0.05 vs. #6 and #7).

**Figure 2:**
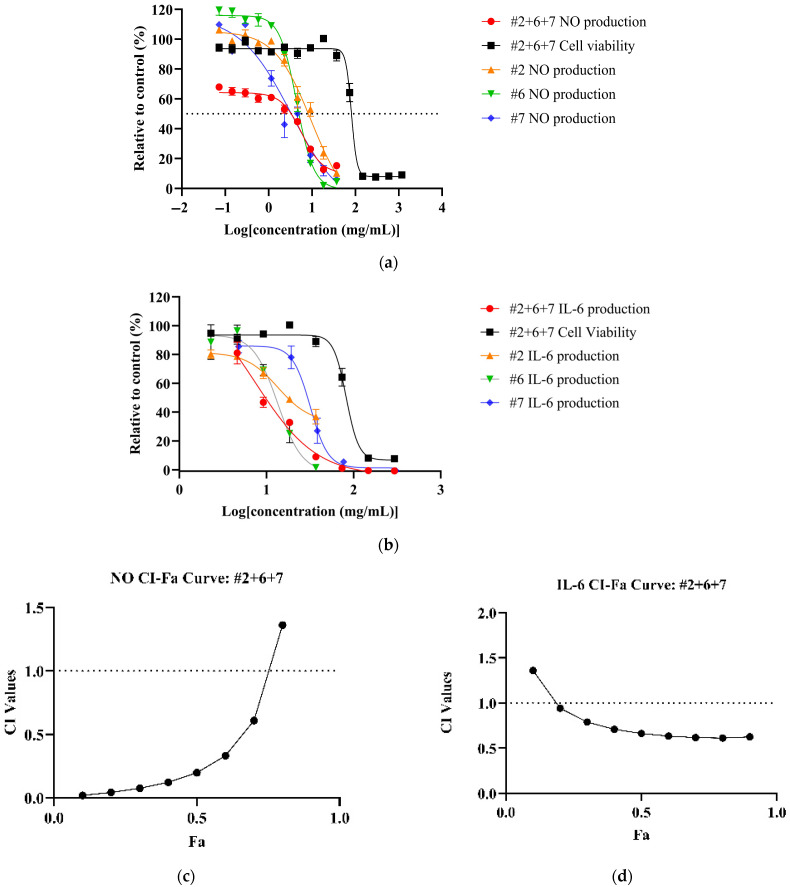
Synergistic effects of bitter orange (#2), mountain pepper berry (#6) and native river mint (#7) to reduce inflammation in LPS-induced RAW 264.7 cells. The figure consists of (**a**) dose–response curves of #2+6+7 for NO production and cell viability in comparison to individual extracts; (**b**) dose–response curves of #2+6+7 for IL-6 expression and cell viability in comparison to individual extracts; (**c**) CI-Fa curve on NO inhibitory effect for #2+6+7; and (**d**) CI-Fa curve on IL-6 inhibitory effect for #2+6+7 with a horizontal dashed line at CI = 1 to denote threshold for interaction.

All NPE combinations demonstrated a dose-dependent reduction of inflammatory markers (See Supplementary Material Figures S7–S9 for dose–response curves for NO, TNF and IL-6 of NPE combinations). Among the combinations, #2+6+7 achieved the most potent reduction of NO (IC_50_: 5.82 ± 2.10 mg/mL) and IL-6 (IC_50_: 7.55 ± 1.58 mg/mL) levels (Figure 2a,b) with corresponding therapeutic indices of 13.91 and 10.72, respectively. Compared to individual extracts, the #2+6+7 combination showed a significantly higher potency than bitter orange (#2) (*p* < 0.05) but not mountain pepper berry (#6) and native river mint (#7). However, for IL-6, the #2+6+7 combination significantly outperformed all the individual extracts (Table 4). For TNF-α, the #6+7 combination yielded the lowest IC_50_ value (48.59 ± 6.78 mg/mL) and highest therapeutic index among the combinations, although it was not as potent as the individual extracts.

CI analysis revealed synergistic effects (CI < 1) for NO and IL-6 inhibition across all NPE combinations. The #2+6+7 combination exhibited the strongest synergy at Fa = 0.5, with the CI values of 0.22 for NO and 0.6 for IL-6 (Figure 2c,d). However, none of the NPE combinations displayed synergistic effects for TNF-α inhibition. (See Supplementary Material Figures S10–S12 for CI-Fa curves for NO, TNF-α and IL-6). Results can be summarised in Table 4.

### 3.4. Synergistic Antioxidant Effects of NPE Combinations

**Figure 3:**
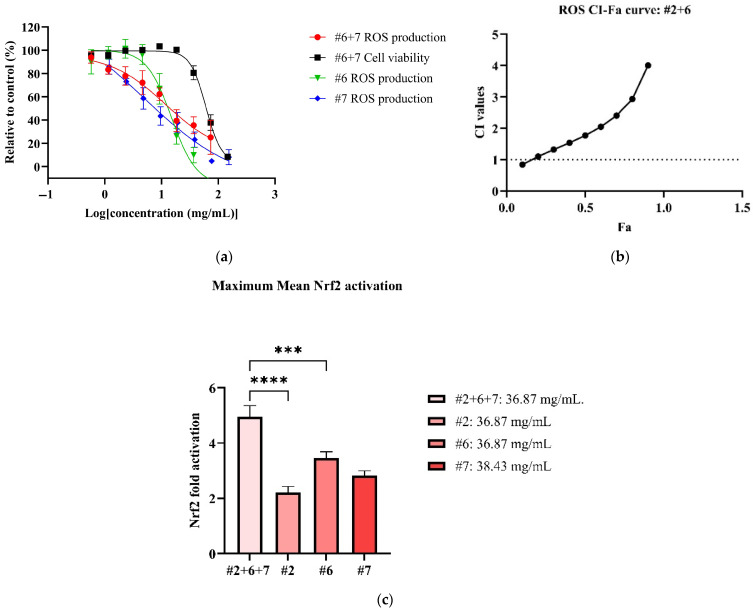
Antioxidant effects of #2, #6 and #7 to reduce ROS and activate Nrf2. Figures consist of (**a**) dose–response curve of #2+6 for ROS in comparison to individual extracts; (**b**) CI-FA curve for ROS inhibitory effect with a horizontal dashed line at CI = 1 to denote threshold for interaction; (**c**) the maximum Nrf2 activation of #2+6+7 compared to #2, #6 and #7. *** *p* < 0.001, **** *p* < 0.0001 vs. #2+6+7. A one-way ANOVA analysis was conducted to identify statistical significance among individual extracts compared to combination #2+6+7.

In the antioxidant cellular models, all NPE combinations inhibited ROS production in LPS-induced RAW 264.7 cells in dose-dependent manner (See Supplementary Material Figure S13). Among the combinations, #6+7 exhibited the most potent ROS inhibition, with an IC_50_ value of 17.98 ± 5.39 mg/mL (Table 5). However, the #6+7 combination was not as potent as its individual extracts (Figure 3a). On the other hand, the #2+6 combination demonstrated the highest therapeutic index (3.35), followed by combinations #6+7 (3.27), #2+7 (2.63) and #2+6+7 (2.10) (Table 5). In CI models (See Supplementary Material Figure S14), no synergistic interaction was detected in all NPE combinations for ROS inhibition, with the exception of weak synergy observed at 0.57 mg/mL in the #2+6 combination (Figure 3b).

All NPE combinations also induced weak Nrf2 activation (See Supplementary Material Figure S15). The #2+6+7 combination produced the highest Nrf2 activation, with a 4.96 ± 1.35-fold increase at non-toxic concentration of 36.87 mg/mL (Figure 3c). The #2+7 combination followed, reaching a significant maximum Nrf2 activation of 4.83 ± 0.51-fold at 18.43 mg/mL (*p* < 0.001 vs. blank). The remaining NPE combinations, #6+7 and #2+7, elicited slightly lower Nrf2 activation of 3.68 ± 0.77-fold and 3.00 ± 0.87-fold, respectively. Notably, the #2+6+7 and #2+7 combinations enhanced Nrf2 activation compared to the individual extracts bitter orange (#2) (2.22 ± 0.79) and mountain pepper berry (#6) (3.47 ± 0.85), with statistical differences (*p* < 0.0001 vs. individual extracts). The results for ROS inhibition and Nrf2 activation are summarised in Table 5.

### 3.5. Synergistic Anti-Inflammatory Effects of NPE Combinations on HDF Skin Cells

The current study assessed the cell viability of LPS-induced HDF cells after treatment of NPE extracts via an Alamar blue assay. The individual extracts reduced cell viability in a dose-dependent manner (See Supplementary Material Figure S16 for dose–response curves of individual extracts). Native river mint (#7) exhibited the lowest LC_50_ value of 2.57 ± 0.05 mg/mL, followed by bitter orange (#2) (LC_50_: 13.71 ± 1.22 mg/mL) and mountain pepper berry (#6) (LC_50_: 16.39 ± 0.94 mg/mL) (Table 6). The LC_50_ values of NPE combinations #2+6+7 and #2+6 were 8.45 ± 0.07 mg/mL and 32.89 ± 2.29 mg/mL, respectively. However, we noted that the #2+6+7 combination reduced cell viability when comparing its LC_50_ values to the #2+6 combination and all the individual extracts.

To evaluate anti-inflammatory activity, the study investigated bitter orange (#2), mountain pepper berry (#6) and native river mint (#7) on LPS-stimulated HDF cells for the IL-6 inhibitory effect. LPS at 50 µg/mL produced 287.50 ± 11.11 pg/mL of IL-6 in HDF cells (*p* < 0.001 vs. blank control: 26.32 ± 5.04 pg/mL).

Among the individual extracts, bitter orange (#2) failed to achieve 50% inhibition of IL-6 production. In contrast, mountain pepper berry (#6) and native river mint (#7) reduced IL-6 levels in a dose-dependent manner, with IC_50_ values of 15.67 ± 3.19 and 4.26 ± 0.42 mg/mL respectively. Only mountain pepper berry (#6) achieved a therapeutic index above 1 (1.04) among the individual extracts.

The study then selected the NPE combinations #2+6 (1:1, *w*/*w*) and #2+6+7 (1:1:1, *w*/*w*/*w*) for further investigation based on previously observed high therapeutic indices for IL-6 inhibition in LPS-induced RAW 264.7 cells (#2+6: 9.18 and #2+6+7: 10.72).

**Figure 4:**
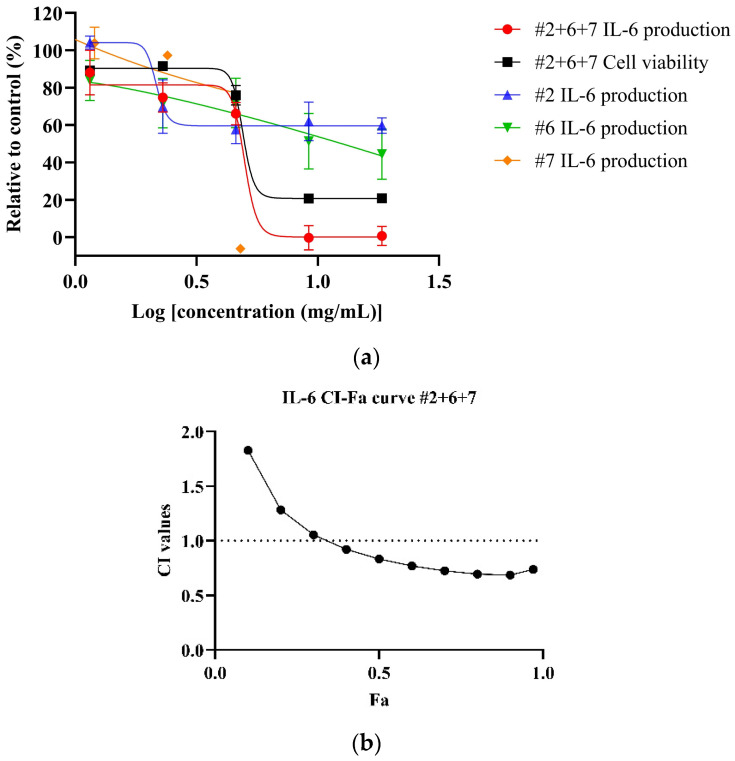

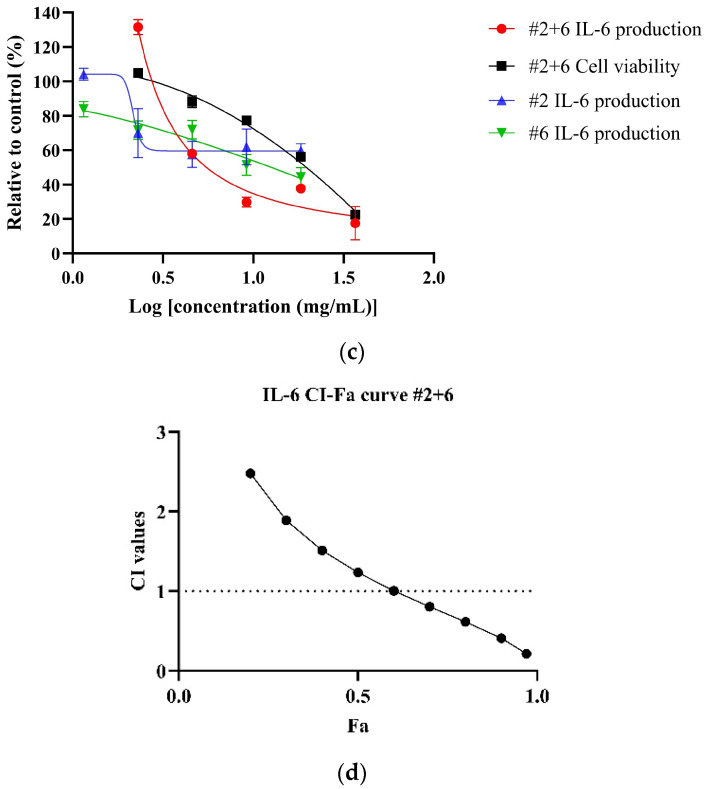
Synergistic effects of #2+6+7 and #2+6 combinations to reduce IL-6 levels in LPS-stimulated HDF cells. Figures include (**a**) dose–response of #2+6+7 on IL-6 inhibition in comparison to individual extracts; (**b**) CI-Fa curve on IL-6 inhibitory effect of #2+6+7 with a horizontal dashed line at CI = 1 to denote threshold for interaction; (**c**) dose–response curves of #2+6 on IL-6 inhibition compared to individual extracts; (**d**) CI-Fa curve on IL-6 inhibitory effect of #2+6 with a horizontal dashed line at CI = 1 to denote threshold for interaction.

Both NPE combinations demonstrated a dose-dependent reduction of IL-6 levels in LPS-stimulated HDF cells. As shown in Figure 4a, the #2+6+7 combination exhibited a greater potency, with an IC_50_ value of 5.18 ± 0.74 mg/mL, compared to the #2+6 combination (Figure 4b) (IC_50_: 9.46 mg/mL) and the individual extracts bitter orange (#2) and mountain pepper berry (#6) (Table 6). CI analysis revealed synergistic interaction in the inhibition of IL-6 in higher concentrations for both the #2+6+7 and #2+6 combinations. Notably, in Figure 4c, the #2+6+7 combination exhibited stronger synergy with a CI value of 0.83 at 50% effect level (Fa = 0.5) for IL-6 inhibition. In contrast, Figure 4d illustrated a CI value greater than 1 at the same effect level for the #2+6 combination. These findings highlight the superior anti-inflammatory potential of the triple combination #2+6+7, particularly in the dermal inflammation model.

### 3.6. Wound Healing Activity of NPE Combinations on HDF Cells

**Figure 5:**
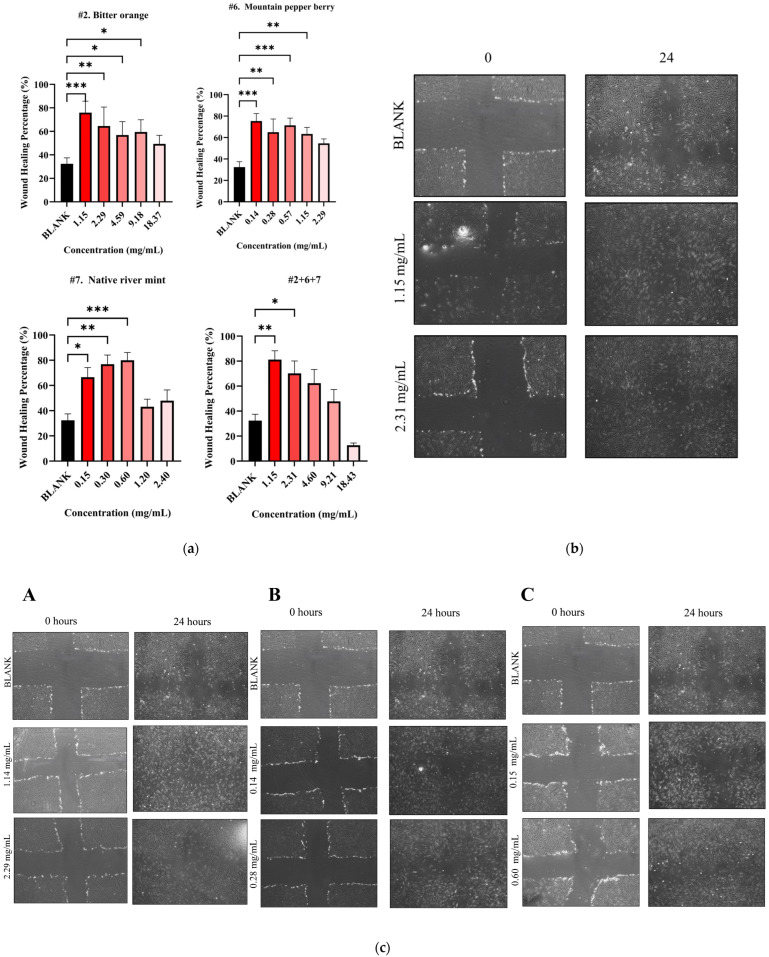
Wound healing activity for NPE combination #2+6+7. (**a**) Wound healing percentage for NPE combination #2+6+7 and their individual extracts; (**b**) microscopic images of potent wound healing activity after 24 h of administering NPE combination #2+6+7 at 2.31 mg/mL and 1.15 mg/mL and (**c**) microscopic images of potent wound healing activity after 24 h of administering individual extracts with two potent concentrations compared to untreated blank control: (**A**) bitter orange at 2.29 mg/mL and 1.14 mg/mL, (**B**) mountain pepper berry at 0.28 mg/mL and 0.14 mg/mL and (**C**) native river mint at 0.60 mg/mL and 0.15 mg/mL. A one-way ANOVA analysis was conducted to identify statistical significance among concentrations compared to blank. * *p* < 0.05, ** *p* < 0.01, *** *p* < 0.001 vs. blank.

The study then subjected both individual and combined NPEs to a wound healing assay using HDF cells. All individual NPEs increased wound healing activity across the tested concentrations compared to the blank control (32.39 ± 5.03%) (Figure 5a). After 24 h, native river mint (#7) at 0.60 mg/mL produced the highest wound healing activity of 79.98 ± 6.11% (*p* < 0.001 vs. blank) as shown in Table 6. Bitter orange (#2) and mountain pepper berry (#6) followed, exhibiting wound healing activities of 75.94 ± 9.73% at 1.15 mg/mL (*p* < 0.001 vs. blank) and 75.39 ± 9.76 mg/mL (*p* < 0.001 vs. blank) at 0.14 mg/mL respectively, after 24 h.

The current study only selected NPE combination #2+6+7 (1:1:1, *w*/*w*) for further evaluation in wound healing assay based on previous synergistic behaviour. This combination at 1.15 mg/mL achieved significant wound healing activity of 81.22 ± 7.05% after 24 h (*p* < 0.01) (Figure 5a). Although the combination demonstrated slightly greater healing compared to individual extracts, no statistical differences were detected by one-way ANOVA analysis. Microscopic images illustrate the wound healing effects for the #2+6+7 combination in Figure 5b and for the individual extracts in Figure 5c.

## 4. Discussion

Combinational therapy via a synergistic approach is a robust method for developing multi-target and multitherapeutic treatments for skin. This can be applied in herbal medicine research when investigating the interactions among whole plant extracts to create safe and effective formulations [57]. Our study revealed for the first time the exploration of using synergistic combinations of Australian native plants for skin protection and wound healing and revealed promising results.

Previous literature reports on the existence of synergy in herbal medicine. As demonstrated by Zhou et al. [47], herbal ingredients ginger (G) and turmeric (T) extracts produce synergistic anti-inflammatory effects. Synergy occurs when two agents exhibit bioactivity via the same pathway, strengthening the signal. Zhou et al. [47] reported that combined G and T synergistically attenuated pro-inflammatory mediators like iNOS, major cytokines (IL-6 and TNF-α) and secondary cytokines (GM-CSF and MCP-1) in LPS and IFN-γ- induced RAW 264.7 cells. These outcomes are due to the significant inhibition of LPS-induced NF-κB p65 translocation, activation of TLR4 and TRAF6, and the phosphorylation of JNK and c-JUN [47]. In our study, we focused on Nrf2 activation as the central mechanism of action. Nrf2 is primarily known to induce antioxidant genes and indirectly modulate the production of inflammatory mediators [58,59]. Our study found that Nrf2 activation was present particularly in bitter orange, mountain pepper berry and native river mint, which also displayed prominent anti-inflammatory and antioxidant activities across inflammatory and oxidative stress cellular models. These findings may highlight the crosstalk relationship between the Nrf2 and NF-κB pathway. The NF-κB pathway is responsible for the production of inflammatory markers that when dysregulated can lead to uncontrollable inflammation. Researchers found that NF-κB and its downstream pro-inflammatory cytokines were enhanced in Nrf2 knockout cells, leading to more aggressive inflammation [60]. Another study found a significant inhibition of NF-κB-DNA-binding activity in diabetic mouse models with the overexpression of Nrf2 [61]. While studies have shown that Nrf2 opposes NF-κB, there is also evidence that NF-κB promotes Nrf2 expressions due to the selective recognition mechanisms of Keap1 with IKKβ or Nrf2 [62]. These effects define the crosstalk relationship between Nrf2 and NF-κB. While the results from the current study may potentially point towards the complex nature of the Nrf2-NF-κB pathway, this study could not investigate these mechanisms for the NPE due to limited time.

The current study investigated combinations of bitter orange (#2), mountain pepper berry (#6) and native river mint (#7) at 1:1 (*w*/*w*) or 1:1:1 (*w*/*w*/*w*) (#2+6; #2+7; #6+7; #2+6+7). Among these combinations, the #2+6+7 combination demonstrated strong synergistic inhibition of NO and IL-6 in LPS-stimulated RAW 264.7. This synergy may be linked to the increase in Nrf2 signalling in #2+6+7 compared to the individual extracts. Synergistic interactions between herbal ingredients may also target different aspects of biological processes. In the current study, we found that the NPE combinations only displayed prominent synergistic activity for anti-inflammatory activity (i.e., NO and IL-6 inhibition) compared to antioxidant activity (i.e., ROS inhibition). This may be attributed to the molecular mechanisms, pathways and target specificity the NPEs may have. For instance, IL-6 and NO production share common signalling pathways such as the activation of NF-κB and the JAK-STAT pathway [63,64,65], whereas generation involves more diverse sources including mitochondrial respiration, NADPH oxidase enzymes and iNOS activity [66]. Thus, ROS inhibition is inherently more complex.

Synergy in our study was also observed in the enhanced cell survival for RAW 264.7 and MCF-7 *ARE*c32 cell lines treated with the NPE combinations. These results indicate that lower dosages in the combinations reduced cytotoxicity [67]. Bitter orange (#2), with its low toxicity, may have contributed to improving the cytotoxic profiles of mountain pepper berry (#6) and native river mint (#7) in synergistic combinations. Hence, future studies should investigate the cell survival mechanisms involved in these effects.

In skin, IL-6 is one of the pleiotropic cytokines synthesised by dermal fibroblasts in response to keratinocyte stimuli, especially IL-1β [68] and leads to skin inflammatory conditions. IL-6 is highly expressed in active psoriatic plaques in patients with psoriasis compared to non-lesional biopsies [69]. In other cases, IL-6 stimulates keratinocyte proliferation, contributing to epidermal hyperplasia [69]. In this study, the optimal NPE combination #2+6+7 showed synergistic interactions for IL-6 inhibitory effect on LPS-induced HDF cells, particularly at higher concentrations. Although the #2+6 combination was more potent overall, the #2+6+7 exhibited greater synergistic interaction at different concentration levels. To our knowledge, this study would be the first to trial and find synergistic combinations of whole plant extracts of Australian native plant species on skin cells.

We also evaluated the wound healing activity of the #2+6+7 combining using HDF cells. Fibroblasts play critical roles throughout the wound healing process. In the inflammatory phase, fibroblasts strengthen the crosstalk relationship between the local immune response and the activation of immune cells to modulate the immune cell recruitment and their behaviour, retention and survival in damaged tissue [70]. Fibroblasts also are crucial for angiogenesis and proliferative phases of wound healing by secreting molecules like fibroblast growth factor, angiopoietin 1, thrombospondin and matrix metalloproteinases [70]. Mechanisms like oxidative stress and excessive inflammation contribute to the persistent non-healing of wounds and prevent wounds from proceeding to the remodelling stage [8]. For example, Deng et al. [71] showed that macrophage-secreted ROS inhibited the migration of cells in prostatic wounds, which was amplified by the MAPK signalling pathway. In our study, the #2+6+7 combination exhibited greater wound healing effects than the individual extracts in HDF cells. Although this study would be the first to report the combined formulation of whole plant extracts #2+6+7 for wound healing activity, the literature supports that the individual plants species have wound healing properties to treat skin conditions [72]. Additionally, our own data confirmed the anti-inflammatory and antioxidant properties of each extract. Overall, our study validates the usefulness of synergistic combinations of the NPE for the potential skin cell protection and wound healing recovery. The synergistic effects observed from our results suggest that these combinations may offer greater therapeutic benefits compared to individual extracts. Furthermore, synergy demonstrates a promising foundation for the development of innovative formulations in cosmetic and dermatological products.

On the other hand, this study recognises that NPEs such as those in the study also carry risks. There is a potential cytotoxicity on skin cells for high doses. However, combining the NPEs may reduce the cytotoxicity and enhance the safety profile. This was partially observed in the current study where LC_50_ values produced by the combined NPE were lower than that of individual components on both RAW 264.7 and HDF cells. However, our focus was primarily on the beneficial anti-inflammatory and antioxidant outcomes and hence the cytotoxicity was based on LC_50_ values of cell viability. This limits the ability to generalise the observed in vitro use to safe topical use. Future studies are warranted to incorporate comprehensive safety assessments such as patch testing and phototoxicity assessments [73,74]. This study also acknowledges some limitations in potentially combining the plant extracts such as antagonistic or additive interactions, which may reduce the efficacy of individual plant extracts [75]. In the current study, we observed an increase in IC_50_ value when bitter orange, mountain pepper berry and native river mint were combined, compared to their individual IC_50_ values, indicating a reduction potency. Other limitations include the complexity of determining which extract from this study was responsible for a particular effect when in combination [76]. Therefore, future studies may include mechanistic studies for inflammatory and oxidative pathways for both individual and combination of plant extracts.

Moreover, the increased complexity and cost of combining extracts should be weighed against therapeutic benefit. While enhanced efficacy demonstrated in the current study may justify use of multi-extract formulation for skin cosmeceutical applications, this study acknowledges that other factors such as formulation stability, regulatory requirements and commercial viability should be considered [77]. Overall, while synergistic combinations offer promise, practical implementations must be informed by thorough preclinical evaluation and cost–benefit analysis.

## 5. Conclusions

Our study found that all NPEs dose-dependently inhibited inflammation in LPS-induced RAW 264.7 cells by reducing inflammatory markers (NO, TNF-α and IL-6). The extracts also exhibited potent antioxidant activity by reducing ROS, selectively scavenged DPPH radicals and activated the Nrf2 pathway. We combined bitter orange (#2), mountain pepper berry (#6) and native river mint (7) at 1:1:1 (*w*/*w*) and found that formulation synergistically reduced LPS-induced NO and IL-6 production in RAW 264.7. This combination also enhanced Nrf2 activation in MCF-7 AREc32 cells and improved cell viability for both MCF-7 AREc32 and RAW 264.7 cells. When further tested on human skin cells, we observed that it synergistically reduced IL-6 production in LPS-induced HDF cells and enhanced wound healing activity. In summary, our findings provide clear evidence that supports the combinations of Australian native plant extracts—bitter orange (#2), mountain pepper berry (#6) and native river mint (7)—acts synergistically to alleviate skin inflammation and promote wound healing.

## Figures and Tables

**Table 1:** Phytochemical constituents in NPEs.

NPE	Compounds
#1: Aniseed myrtle	Procyanidins, tryptophan, catechin, myricetin, quercetin, anethole, flavonoid glycosides, flavan-3-ol (flavanols), ellagic acid, amino acids
#2: Bitter orange	Naringin, hesperidin, nobiletin, flavone derivatives, amino acids, phenolics
#3: Blue butterfly pea	Anthocyanins, adenosine, nucleotides, kaempferol, quercetin, rutin, lignan, phenolics, flavonoid glycosides, amino acids
#4: Blue cypress leaf	Gallocatechin, procyanidins, catechin, hypolaetin, tetrahydroxy flavone, flavonoid glucosides, amino acids, organic acids, sugars, phenolics
#5: Emu bush	Chlorogenic acid, ferulic acid, quercetin, vebrascoside, flavone glycoside, isorhamnetin, phenolics, caffeic acid ester derivatives
#6: Mountain pepper berry	Anthocyanins, polygodial, protocatechuic acid, chlorogenic acid, rutin, quercetin, flavonoid glycosides, sugars, amino acids, organic acids, phenolics
#7: Native river mint	Rosmarinic acid, methoxy-flavones, flavonoid glycosides, terpenoids, amino acids, organic acids, phenolics
#8: Pineapple	Polyamines (spermine/spermidine), glutamyl-cysteine and derivatives, tryptophan sinapic acid and derivatives, phenolics, amino acids, sugars, organic acids
#9: Kakadu plum	Natural vitamin c (abscorbic acid), gallic acid, ellagic acid, amino acids, tryptophan, flavone c-glycoside

**Table 2:** Anti-inflammatory activity, toxicity and therapeutic indices of the nine NPE in inhibiting LPS-induced NO, TNF-α and IL-6 in RAW 264.7 cells. (n ≥ 3 experiments). Data shown as mean ± SEM.

NPE	Cell Viability LC_50_ (mg/mL)	NO		TNF-α		IL-6	
		IC_50_ (mg/mL)	Therapeutic Index	IC_50_ (mg/mL)	Therapeutic Index	IC_50_ (mg/mL)	Therapeutic Index
#1: Aniseed myrtle	19.78 ± 4.25	1.11 ± 0.40	17.80	4.38 ± 2.12	4.50	3.42 ± 1.42	5.78
#2: Bitter orange	>100	7.44 ± 2.93	28.17	15.70 ± 1.70	13.34	13.55 ± 4.02	15.47
#3: Blue butterfly pea	19.33 ± 3.24	17.37 ± 3.34	1.11	29.5 ± 10.01	<1	58.68 ± 9.37	<1
#4: Blue cypress leaf	81.12 ± 6.04	22.02 ± 3.13	3.68	89.26 ± 14.20	<1	88.05 ± 6.08	<1
#5: Emu bush	90.19 ± 8.30	12.09 ± 4.21	7.4	185.10	<1	99.03 ± 14.23	<1
#6: Mountain pepper berry	46.74 ± 5.52	4.44 ± 0.51	10.53	13.32 ± 3.22	3.51	13.49 ± 6.83	3.46
#7: Native river mint	56.70 ± 5.72	3.13 ± 1.31	18.12	23.42 ± 2.23	2.42	31.88 ± 5.43	1.77
#8: Pineapple	>100	73.03 ± 11.87	1.97	134.40 ± 15.30	1.07	87.48 ± 5.67	1.64
#9: Kakadu plum	65.64 ± 11.63	15.02 ± 4.31	4.30	110.20 ± 12.60	<1	73.84 ± 3.10	<1

**Table 3:** Antioxidant activity, toxicity and therapeutic index of the nine NPEs for DPPH scavenging activity, inhibition of LPS-induced ROS in RAW 264.7 cells, and Nrf2 activation in AREc32 cells (n ≥ 3 experiments). Data shown as mean ± SEM.

NPE	DPPH	ROS		Nrf2 Activation		
	IC_50_ (mg/mL)	IC_50_ (mg/mL)	Therapeutic Index	Concentration with the Maximum Nrf2 Activation (mg/mL)	Maximum Mean Nrf2 Fold Increase	Cell Viability for MC-7 AREc32 Cells at Maximum Nrf2 Activation (%)
#1: Aniseed myrtle	1.85 ± 0.71	3.21 ± 0.76	6.16	9.76	1.62 ± 0.75	92.90 ± 2.66
#2: Bitter orange	2.08 ± 0.34	9.69 ± 2.82	21.63	36.87	2.22 ± 0.79	94.86 ± 8.27
#3: Blue butterfly pea	204.99 ± 42.65	27.05 ± 5.40	<1	9.22	0.86 ± 0.21	82.25 ± 6.48
#4: Blue cypress leaf	64.31 ± 10.90	29.87 ± 7.73	2.71	75	1.42 ± 0.78	67.59 ± 6.52
#5: Emu bush	1081.35 ± 46.58	14.82 ± 3.31	6.09	37.81	1.67 ± 0.37	88.63 ± 4.02
#6: Mountain pepper berry	0.95 ± 0.06	6.56 ± 1.04	7.13	36.87	3.47 ± 0.85	92.39 ± 6.60
#7: Native river mint	9.65 ± 0.37	4.95 ± 1.50	11.45	38.43	2.82 ± 0.60	92.33 ± 4.27
#8: Pineapple	1112.01 ± 17.89	>100	<1	10.31	1.74 ± 0.59	96.89 ± 2.91
#9: Kakadu plum	576.03 ± 3.35	16.70 ± 5.78	3.90	36.56	1.61 ± 0.29	92.32 ± 2.16

**Table 4:** Anti-inflammatory, toxicity, therapeutic index and synergistic interaction of the NPE combinations (1:1, 1:1:1 *w*/*w*) in inhibiting LPS-induced NO, TNF-α and IL-6 in LPS-induced RAW 264.7 cells (n ≥ 3 experiments). Data shown as mean ± SEM.

NPE	Cell Viability LC_50_ (mg/mL)	NO			TNF-α			IL-6		
		IC_50_ (mg/mL)	Therapeutic Index	CI Value Fa = 0.5	IC_50_ (mg/mL)	Therapeutic Index	CI Value Fa = 0.5	IC_50_ (mg/mL)	Therapeutic Index	CI Value Fa = 0.5
COMBINATION										
#2+6	72. 85 ± 7.65 ^#^	6.70 ± 3.75 *	10.87	0.23	55.44 ± 13.68 *	1.31	>1	7.93 ± 1.54 *	9.18	0.94
#2+7	91.81 ± 9.34 ^∆^	10.68 ± 0.11 *	8.59	0.24	78.56 ± 16.17 *	1.16	>1	37.68 ± 12.23	2.43	0.87
#6+7	58.96 ± 5.02 ^∆^	6.41 ± 2.32 *	9.20	0.19	48.59 ± 6.78	1.21	>1	27.11 ± 1.14	2.17	>1
#2+6+7	80.97 ± 3.76 ^#∆^	5.82 ± 2.10 *^#∆^	13.91	0.20	78.56 ± 9.49	1.03	>1	7.55 ± 1.58 ^∆^	10.72	0.66
INDIVIDUAL										
#2: Bitter orange	>100 ^#^	7.44 ± 2.93 ^∆^	28.17	N/A	15.70 ± 1.70 ^∆^	13.34	N/A	13.55 ± 4.02	15.47	N/A
#6: Mountain pepper berry	46.74 ± 5.52 *	4.44 ± 0.51	10.50	N/A	13.32 ± 3.22	3.51	N/A	13.49 ± 6.83	3.46	N/A
#7: Native river mint	56.70 ± 11.44 *	3.13 ± 1.31 *	18.12	N/A	23.42 ± 2.23 *	2.42	N/A	31.88 ± 5.43	1.77	N/A

* *p* < 0.05 when compared to NPE #2, ^#^ *p* < 0.05 when compared to NPE #6, ^∆^ *p* < 0.05 when compared to NPE #7 using one-way ANOVA.

**Table 5:** Antioxidant, toxicity, therapeutic index and synergistic interaction of the NPE combinations (1:1, 1:1:1 *w*/*w*) in LPS-induced ROS in RAW 264.7 cells and activation of Nrf2 in MC-7AREc32 (n ≥ 3 experiments). Data shown as mean ± SEM.

NPE	Cell Viability LC_50_ (mg/mL)	ROS			Nrf2 Activation		
		IC_50_ (mg/mL)	Therapeutic Index	CI Value Fa = 0.5	Concentration with the Maximum Nrf2 Activation (mg/mL)	Maximum Mean Nrf2 Fold Increase	Cell Viability (MC-7 AREc32) at Maximum Nrf2 Activation (%)
COMBINATION							
#2+6	72. 85 ± 7.65	21.74 ± 6.73	3.35	>1	36.87	3.00 ± 0.87	91.24 ± 8.34
#2+7	91.81± 9.34	34.79 ± 7.40	2.63	>1	18.43	4.83 ± 0.51 *^#^	95.95 ± 9.22
#6+7	58.96 ± 5.02	17.98 ± 5.39	3.27	>1	36.87	3.68 ± 0.77 *	95.65 ± 5.16
#2+6+7	80.97 ± 3.76	38.50 ± 7.58 *^#∆^	2.10	>1	36.87	4.96 ± 1.35 *^#^	101.23 ± 7.76
INDIVIDUAL							
#2: Bitter orange	>100	9.69 ± 2.82	21.63	N/A	36.87	2.22 ± 0.79 ^#^	94.86 ± 8.27
#6: Mountain pepper berry	46.74 ± 5.52	6.56 ± 1.04	7.13	N/A	36.87	3.47 ± 0.85 *	92.39 ± 6.60
#7: Native river mint	56.70 ± 11.44	4.95 ± 1.50	11.45	N/A	38.43	2.82 ± 0.60	92.33 ± 4.27

* *p* < 0.05 when compared to NPE #2, ^#^ *p* < 0.05 when compared to NPE #6, ^∆^ *p* < 0.05 when compared to NPE #7 using one-way ANOVA.

**Table 6:** IL-6 inhibitory effect and wound healing activity of individual and combined NPE extracts bitter orange (#2), mountain pepper berry (#6) and native river mint (#7) on HDF cells (n ≥ 3 experiments). Data are shown as mean ± SEM.

NPE	LC_50_ (mg/mL)	IL-6			Wound Healing	
		IC_50_ (mg/mL)	Therapeutic Index	CI Values When Fa = 0.5	Concentration (mg/mL) with Highest Wound Healing Activity	Wound Healing Percentage (%)
COMBINATION						
#2+6	32.89 ± 2.29	9.46	3.48	>1	N/A	N/A
#2+6+7	8.45 ± 0.07	5.18 ± 0.74 ^#^	1.63	0.83	1.15	81.22 ± 7.05
INDIVIDUAL						
#2. Bitter orange	13.71 ± 1.22	>18.37 ^#^	<1	N/A	1.15	75.94 ± 9.73
#6. Mountain pepper berry	16.39 ± 0.94	15.67 ± 3.19 *^∆^	1.04	N/A	0.14	75.39 ± 9.76
#7. Native river mint	2.57 ± 0.05	4.26 ± 0.42 ^#^	<1	N/A	0.60	79.98 ± 6.11

* *p* < 0.05 when compared to NPE extract #2, ^#^ *p* < 0.05 when compared to NPE extract #6, ^∆^ *p* < 0.05 when compared to NPE extract #7 using one-way ANOVA.

## Data Availability

The original contributions presented in this study are included in the article/Supplementary Material. Further inquiries can be directed to the corresponding author.

## Supplementary Materials

The following supporting information can be downloaded at https://www.mdpi.com/article/10.3390/biomedicines13071754/s1, The supporting materials for this study are as follows: Table S1: NPE Product information, Figure S1: Dose–response curves of individual NPEs for NO, Figure S2: Dose–response curves of individual NPEs for TNF-α, Figure S3: Dose–response curves of individual NPEs for IL-6, Figure S4: DPPH scavenging effect for individual NPEs, Figure S5: Dose–response curves of individual NPEs for ROS, Figure S6: Nrf2 fold activation of individual NPEs, Figure S7: Dose–response curves of combined NPE for NO, Figure S8: Dose–response curves of combined NPEs for TNF-α, Figure S9: Dose–response curves of combined NPEs for IL-6, Figure S10: CI-Fa curves of combined NPE for NO, Figure S11: CI-Fa curves of combined NPEs for TNF-α, Figure S12: CI-Fa curves of combined NPEs for IL-6, Figure S13: Dose–response curves of combined NPEs for ROS, Figure S14: CI-Fa curves of combined NPEs for ROS, Figure S15: Nrf2 fold activation of combined NPEs, Figure S16: Dose–response curves of individual NPEs for IL-6 in HDF.

## References

[1] Brand R.M., Wipf P., Durham A., Epperly M.W., Greenberger J.S., Falo L.D. (2018). Targeting Mitochondrial Oxidative Stress to Mitigate UV-Induced Skin Damage. Front. Pharmacol..

[2] Ansary T.M., Hossain M.R., Kamiya K., Komine M., Ohtsuki M. (2021). Inflammatory Molecules Associated with Ultraviolet Radiation-Mediated Skin Aging. Int. J. Mol. Sci..

[3] Liu H.-M., Cheng M.-Y., Xun M.-H., Zhao Z.-W., Zhang Y., Tang W., Cheng J., Ni J., Wang W. (2023). Possible Mechanisms of Oxidative Stress-Induced Skin Cellular Senescence, Inflammation, and Cancer and the Therapeutic Potential of Plant Polyphenols. Int. J. Mol. Sci..

[4] Wagener F., Carels C., Lundvig D. (2013). Targeting the Redox Balance in Inflammatory Skin Conditions. Int. J. Mol. Sci..

[5] Shen G.X. (2010). Oxidative stress and diabetic cardiovascular disorders: Roles of mitochondria and NADPH oxidase. Can. J. Physiol. Pharmacol..

[6] De Jager T.L., Cockrell A.E., Du Plessis S.S. (2017). Ultraviolet Light Induced Generation of Reactive Oxygen Species.

[7] Wei M., He X., Liu N., Deng H. (2024). Role of reactive oxygen species in ultraviolet-induced photodamage of the skin. Cell Div..

[8] Dong Y., Wang Z. (2023). ROS-scavenging materials for skin wound healing: Advancements and applications. Front. Bioeng. Biotechnol..

[9] Son Y., Cheong Y.-K., Kim N.-H., Chung H.-T., Kang D.G., Pae H.-O. (2011). Mitogen-Activated Protein Kinases and Reactive Oxygen Species: How Can ROS Activate MAPK Pathways?. J. Signal Transduct..

[10] Shin J.-W., Kwon S.-H., Choi J.-Y., Na J.-I., Huh C.-H., Choi H.-R., Park K.-C. (2019). Molecular Mechanisms of Dermal Aging and Antiaging Approaches. Int. J. Mol. Sci..

[11] Xu F., Xu J., Xiong X., Deng Y. (2019). Salidroside inhibits MAPK, NF-κB, and STAT3 pathways in psoriasis-associated oxidative stress via SIRT1 activation. Redox Rep..

[12] Fu J., Zeng Z., Zhang L., Wang Y., Li P. (2020). 4′-O-β-D-glucosyl-5-O-methylvisamminol ameliorates imiquimod-induced psoriasis-like dermatitis and inhibits inflammatory cytokines production by suppressing the NF-κB and MAPK signaling pathways. Braz. J. Med. Biol. Res..

[13] Ngoc L.T.N., Tran V.V., Moon J.-Y., Chae M., Park D., Lee Y.-C. (2019). Recent Trends of Sunscreen Cosmetic: An Update Review. Cosmetics.

[14] Chong M., Fonacier L. (2016). Treatment of Eczema: Corticosteroids and Beyond. Clin. Rev. Allergy Immunol..

[15] Del Grossi Moura M., Cruz Lopes L., Silva M.T., Barberato-Filho S., Motta R.H.L., Bergamaschi C.C. (2018). Use of steroid and nonsteroidal anti-inflammatories in the treatment of rheumatoid arthritis: Systematic review protocol. Medicine.

[16] Uva L., Miguel D., Pinheiro C., Antunes J., Cruz D., Ferreira J., Filipe P. (2012). Mechanisms of Action of Topical Corticosteroids in Psoriasis. Int. J. Endocrinol..

[17] Shi C., Ye Z., Shao Z., Fan B., Huang C., Zhang Y., Kuang X., Miao L., Wu X., Zhao R. (2023). Multidisciplinary Guidelines for the Rational Use of Topical Non-Steroidal Anti-Inflammatory Drugs for Musculoskeletal Pain (2022). J. Clin. Med..

[18] Llamas-Velasco M., Garcia-Diez A. (2010). Climate Change and Skin: Diagnostic and Therapeutic. Chall. Dermo-Sifiograficas.

[19] Egambaram O.P., Kesavan Pillai S., Ray S.S. (2020). Materials Science Challenges in Skin UV Protection: A Review. Photochem. Photobiol..

[20] Meena S., Gupta L.K., Khare A.K., Balai M., Mittal A., Mehta S., Bhatri G. (2017). Topical Corticosteroids Abuse: A Clinical Study of Cutaneous Adverse Effects. Indian J. Dermatol..

[21] Yu S.H., Drucker A.M., Lebwohl M., Silverberg J.I. (2018). A systematic review of the safety and efficacy of systemic corticosteroids in atopic dermatitis. J. Am. Acad. Dermatol..

[22] Paiva J.P., Diniz R.R., Leitão A.C., Cabral L.M., Fortunato R.S., Santos B.A.M.C., de Padula M. (2020). Insights and controversies on sunscreen safety. Crit. Rev. Toxicol..

[23] Sander M., Sander M., Burbidge T., Beecker J. (2020). The efficacy and safety of sunscreen use for the prevention of skin cancer. Can. Med. Assoc. J..

[24] Phan K., Smith S.D. (2021). Topical corticosteroids and risk of diabetes mellitus: Systematic review and meta-analysis. J. Dermatol. Treat..

[25] Zhou X., Seto S.W., Chang D., Kiat H., Razmovski-Naumovski V., Chan K., Bensoussan A. (2016). Synergistic Effects of Chinese Herbal Medicine: A Comprehensive Review of Methodology and Current Research. Front. Pharmacol..

[26] Lehár J., Krueger A.S., Avery W., Heilbut A.M., Johansen L.M., Price E.R., Rickles R.J., Iii G.F.S., E Staunton J., Jin X. (2009). Synergistic drug combinations tend to improve therapeutically relevant selectivity. Nat. Biotechnol..

[27] Amparo T.R., Seibert J.B., Vieira P.M.A., Teixeira L.F.M., Santos O., de Souza G.H.B. (2020). Herbal medicines to the treatment of skin and soft tissue infections: Advantages of the multi-targets action. Phytother. Res..

[28] Ahuja A., Gupta J., Gupta R. (2021). Miracles of Herbal Phytomedicines in Treatment of Skin Disorders: Natural Healthcare Perspective. Infect. Disord. Drug Targets.

[29] Konczak I., Zabaras D., Dunstan M., Aguas P. (2010). Antioxidant capacity and phenolic compounds in commercially grown native Australian herbs and spices. Food Chem..

[30] Uddin A.B.M.N., Hossain F., Reza A.S.M.A., Nasrin M.S., Alam A.H.M.K. (2022). Traditional uses, pharmacological activities, and phytochemical constituents of the genus Syzygium: A review. Food Sci. Nutr..

[31] Guo Y., Sakulnarmrat K., Konczak I. (2014). Anti-inflammatory potential of native Australian herbs polyphenols. Toxicol. Rep..

[32] Alqahtani A., Abdelhameed M.F., Abdou R., Ibrahim A.M., Dawoud M., Alasmari S.M., El Raey M.A., Attia H.G. (2023). Mechanistic action of linalyl acetate: Acyclic monoterpene isolated from bitter orange leaf as anti-inflammatory, analgesic, antipyretic agent: Role of TNF-α, IL1β, PGE2, and COX-2. Ind. Crops Prod..

[33] Kang S.R., Park K.I., Park H.S., Lee D.H., Kim J.A., Nagappan A., Kim E.H., Lee W.S., Shin S.C., Park M.K. (2011). Anti-inflammatory effect of flavonoids isolated from Korea Citrus aurantium L. on lipopolysaccharide-induced mouse macrophage RAW 264.7 cells by blocking of nuclear factor-kappa B (NF-κB) and mitogen-activated protein kinase (MAPK) signalling pathways. Food Chem..

[34] Jeyaraj E.J., Lim Y.Y., Choo W.S. (2021). Extraction methods of butterfly pea (*Clitoria ternatea*) flower and biological activities of its phytochemicals. J. Food Sci. Technol..

[35] Zakaria N.N.A., Okello E.J., Howes M.J., Birch-Machin M.A., Bowman A. (2018). In vitro protective effects of an aqueous extract of *Clitoria ternatea* L. flower against hydrogen peroxide-induced cytotoxicity and UV-induced mtDNA damage in human keratinocytes. Phytother. Res..

[36] Nair V., Bang W.Y., Schreckinger E., Andarwulan N., Cisneros-Zevallos L. (2015). Protective Role of Ternatin Anthocyanins and Quercetin Glycosides from Butterfly Pea (*Clitoria ternatea* Leguminosae) Blue Flower Petals against Lipopolysaccharide (LPS)-Induced Inflammation in Macrophage Cells. J. Agric. Food Chem..

[37] Ololade Z., No O. (2013). Recovered Secondary Metabolites of Post-Hydrodistilled Callitris columellaris Leaf and their Free Radical Scavenging Potentials. Org. Chem. Curr. Res..

[38] Ololade Z.S.O.O., Kolawole S., Onipede O.J. (2012). Phyto-chemicals, Free Radical Scavenging and Anti-inflammatory Activity of the Leaf Essential Oil of *Callitris columellaris* F. Meull from Plateau Sate, Nigeria. Int. J. Appl. Res. Technol..

[39] Cock I.E., Baghtchedjian L., Cordon M.-E., Dumont E. (2022). Phytochemistry, Medicinal Properties, Bioactive Compounds, and Therapeutic Potential of the Genus Eremophila (Scrophulariaceae). Molecules.

[40] Sadgrove N.J., Jones G.L. (2013). A possible role of partially pyrolysed essential oils in Australian Aboriginal traditional ceremonial and medicinal smoking applications of *Eremophila longifolia* (R. Br.) F. Muell (Scrophulariaceae). J. Ethnopharmacol..

[41] Cock I. (2013). The phytochemistry and chemotherapeutic potential of *Tasmannia lanceolata* (Tasmanian pepper): A review. Pharmacogn. Commun..

[42] Tang K.S., Konczak I., Zhao J. (2016). Identification and quantification of phenolics in Australian native mint (*Mentha australis R. Br*.). Food Chem..

[43] Ajagun-Ogunleye M.O., Ebuehi O.A.T. (2020). Evaluation of the anti-aging and antioxidant action of *Ananas sativa* and *Moringa oleifera* in a fruit fly model organism. J. Food Biochem..

[44] Hossain M.A., Rahman S.M.M. (2011). Total phenolics, flavonoids and antioxidant activity of tropical fruit pineapple. Food Res. Int..

[45] Li J., Mao B., Tang X., Zhang Q., Zhao J., Zhang H., Cui S. (2023). Protective Effects of Naringenin and Apigenin in Ameliorating Skin Damage via Mediating the Nrf2 and NF-κB Pathways in Mice. Foods.

[46] Tan A.C., Konczak I., Ramzan I., Sze D.M.Y. (2011). Antioxidant and cytoprotective activities of native Australian fruit polyphenols. Food Res. Int..

[47] Zhou X., Munch G., Wohlmuth H., Afzal S., Kao M.T., Al-Khazaleh A., Low M., Leach D., Li C.G. (2022). Synergistic Inhibition of Pro-Inflammatory Pathways by Ginger and Turmeric Extracts in RAW 264.7 Cells. Front. Pharmacol..

[48] Afzal S., Zhou X., Or K., Raju R., Munch G. (2023). Identification of Nrf2 Activators from the Roots of *Valeriana officinalis*. Planta Medica.

[49] Dixon K.M., Deo S.S., Norman A.W., Bishop J.E., Halliday G.M., Reeve V.E., Mason R. (2007). In vivo relevance for photoprotection by the vitamin D rapid response pathway. J. Steroid Biochem. Mol. Biol..

[50] Baliyan S., Mukherjee R., Priyadarshini A., Vibhuti A., Gupta A., Pandey R.P., Chang C.-M. (2022). Determination of Antioxidants by DPPH Radical Scavenging Activity and Quantitative Phytochemical Analysis of Ficus religiosa. Molecules.

[51] Phukan K., Devi R., Chowdhury D. (2022). Insights into Anti-Inflammatory Activity and Internalization Pathway of Onion Peel-Derived Gold Nano Bioconjugates in RAW 264.7 Macrophages. ACS Omega.

[52] Zagorski J.W., Turley A.E., Dover H.E., Vandenberg K.R., Compton J.R., Rockwell C.E. (2013). The Nrf2 Activator, tBHQ, Differentially Affects Early Events Following Stimulation of Jurkat Cells. Toxicol. Sci..

[53] Tan W.S., Arulselvan P., Ng S.F., Taib C.N.M., Sarian M.N., Fakurazi S. (2020). Healing Effect of Vicenin-2 (VCN-2) on Human Dermal Fibroblast (HDF) and Development VCN-2 Hydrocolloid Film Based on Alginate as Potential Wound Dressing. Biomed. Res. Int..

[54] Zhou X., Razmovski-Naumovski V., Kam A., Chang D., Li C., Bensoussan A., Chan K. (2017). Synergistic Effects of Danshen (Salvia Miltiorrhizae Radix et Rhizoma) and Sanqi (Notoginseng Radix et Rhizoma) Combination in Angiogenesis Behavior in EAhy 926 Cells. Medicines.

[55] Suarez-Arnedo A., Torres Figueroa F., Clavijo C., Arbeláez P., Cruz J.C., Muñoz-Camargo C. (2020). An image J plugin for the high throughput image analysis of in vitro scratch wound healing assays. PLoS ONE.

[56] Chou T.-C. (2018). The combination index (CI < 1) as the definition of synergism and of synergy claims. Synergy.

[57] Yang Y., Zhang Z., Li S., Ye X., Li X., He K. (2014). Synergy effects of herb extracts: Pharmacokinetics and pharmacodynamic basis. Fitoterapia.

[58] Gęgotek A., Skrzydlewska E. (2015). The role of transcription factor Nrf2 in skin cells metabolism. Arch. Dermatol. Res..

[59] Ahmed S.M., Luo L., Namani A., Wang X.J., Tang X. (2017). Nrf2 signaling pathway: Pivotal roles in inflammation. Biochim. Biophys. Acta Mol. Basis Dis..

[60] Mohamed E.E., Ahmed O.M., Abdel-Moneim A., Zoheir K.M.A., Elesawy B.H., Al Askary A., Hassaballa A., El-Shahawy A.A.G. (2022). Protective Effects of Naringin-Dextrin Nanoformula against Chemically Induced Hepatocellular Carcinoma in Wistar Rats: Roles of Oxidative Stress, Inflammation, Cell Apoptosis, and Proliferation. Pharmaceuticals.

[61] Song M.Y., Kim E.K., Moon W.S., Park J.W., Kim H.J., So H.S., Park R., Kwon K.B., Park B.H. (2009). Sulforaphane protects against cytokine- and streptozotocin-induced beta-cell damage by suppressing the NF-kappaB pathway. Toxicol. Appl. Pharmacol..

[62] Gao W., Guo L., Yang Y., Wang Y., Xia S., Gong H., Zhang B.-K., Yan M. (2022). Dissecting the Crosstalk Between Nrf2 and NF-κB Response Pathways in Drug-Induced Toxicity. Front. Cell Dev. Biol..

[63] Brasier A.R. (2010). The nuclear factor- B-interleukin-6 signalling pathway mediating vascular inflammation. Cardiovasc. Res..

[64] Arias-Salvatierra D., Silbergeld E.K., Acosta-Saavedra L.C., Calderon-Aranda E.S. (2011). Role of nitric oxide produced by iNOS through NF-kappaB pathway in migration of cerebellar granule neurons induced by Lipopolysaccharide. Cell Signal.

[65] Huang B., Lang X., Li X. (2022). The role of IL-6/JAK2/STAT3 signaling pathway in cancers. Front. Oncol..

[66] Canton M., Sanchez-Rodriguez R., Spera I., Venegas F.C., Favia M., Viola A., Castegna A. (2021). Reactive Oxygen Species in Macrophages: Sources and Targets. Front. Immunol..

[67] Hu C., Mi W., Li F., Zhu L., Ou Q., Li M., Li T., Ma Y., Zhang Y., Xu Y. (2024). Optimizing drug combination and mechanism analysis based on risk pathway crosstalk in pan cancer. Sci. Data.

[68] Duncan M.R., Berman B. (1991). Stimulation of collagen and glycosaminoglycan production in cultured human adult dermal fibroblasts by recombinant human interleukin 6. J. Investig. Dermatol..

[69] Grossman R.M., Krueger J., Yourish D., Granelli-Piperno A., Murphy D.P., May L.T., Kupper T.S., Sehgal P.B., Gottlieb A.B. (1989). Interleukin 6 is expressed in high levels in psoriatic skin and stimulates proliferation of cultured human keratinocytes. Proc. Natl. Acad. Sci. USA.

[70] Cialdai F., Risaliti C., Monici M. (2022). Role of fibroblasts in wound healing and tissue remodeling on Earth and in space. Front. Bioeng. Biotechnol..

[71] Deng Z., Shi F., Zhou Z., Sun F., Sun M.-H., Sun Q., Chen L., Li D., Jiang C.-Y., Zhao R.-Z. (2019). M1 macrophage mediated increased reactive oxygen species (ROS) influence wound healing via the MAPK signaling in vitro and in vivo. Toxicol. Appl. Pharmacol..

[72] Mani J.S., Johnson J.B., Hosking H., Ashwath N., Walsh K.B., Neilsen P.M., Broszczak D.A., Naiker M. (2021). Antioxidative and therapeutic potential of selected Australian plants: A review. J. Ethnopharmacol..

[73] Kim K., Park H., Lim K.-M. (2015). Phototoxicity: Its Mechanism and Animal Alternative Test Methods. Toxicol. Res..

[74] Aalto-Korte K., Suuronen K., Frosch P.J. (2019). Patch Testing with the Patients’ Own Products.

[75] Donkor M.N., Donkor A.-M., Mosobil R. (2023). Combination therapy: Synergism among three plant extracts against selected pathogens. BMC Res. Notes.

[76] El Kahlout K.E.M., El Borsh W., Aksoy A., El Kichaoi A.Y., El Hindi M.W., El Ashgar N.M. (2020). Evaluation of Antibacterial and Synergistic/Antagonistic Effect of Some Medicinal Plants Extracted by Microwave and Conventional Methods. J. Biosci. Med..

[77] Millward H., Lewis A. (2005). Barriers to successful new product development within small manufacturing companies. J. Small Bus. Enterp. Dev..

