# Supplementary material for: Synergistic Combinations of Native Australian Plants For Skin Inflammation and Wound Healing
Source: Biomedicines. 2025 Jul 17;13(7):1754. doi: 10.3390/biomedicines13071754 (PMC12293028; doi:10.3390/biomedicines13071754)
Supplement: Supplementary file 1 [file biomedicines-13-01754-s001.zip › biomedicines-3700117-supplementary.pdf]

Table S1. NPE product details and estimated original concentrations (g/mL).

Product Number	Batch Number	Common English Name of the NPE	Code for the NPE	Estimated Original Concentrations (g/mL)
ANE0028SB	NE220112-03	Aniseed Myrtle	#1	1.25
ANE0126SB	NE210260-11	Bitter Orange	#2	1.18
ANE0615SB	NE220307-11	Blue Butterfly Pea	#3	1.18
ANE0057SB	NE220251-09	Blue Cypress Leaf	#4	1.20
ANE0039SB	NE220251-09	Emu Bush	#5	1.21
ANE0036SB	NE210026-04	Mountain Pepper Berry	#6	1.18
ANE0125SB	NE22096-11	Native River Mint	#7	1.23
ANE0510SB	NE220077-02	Pineapple	#8	1.32
ANE0013SB	NE220253-09	Kakadu Plum	#9	1.17

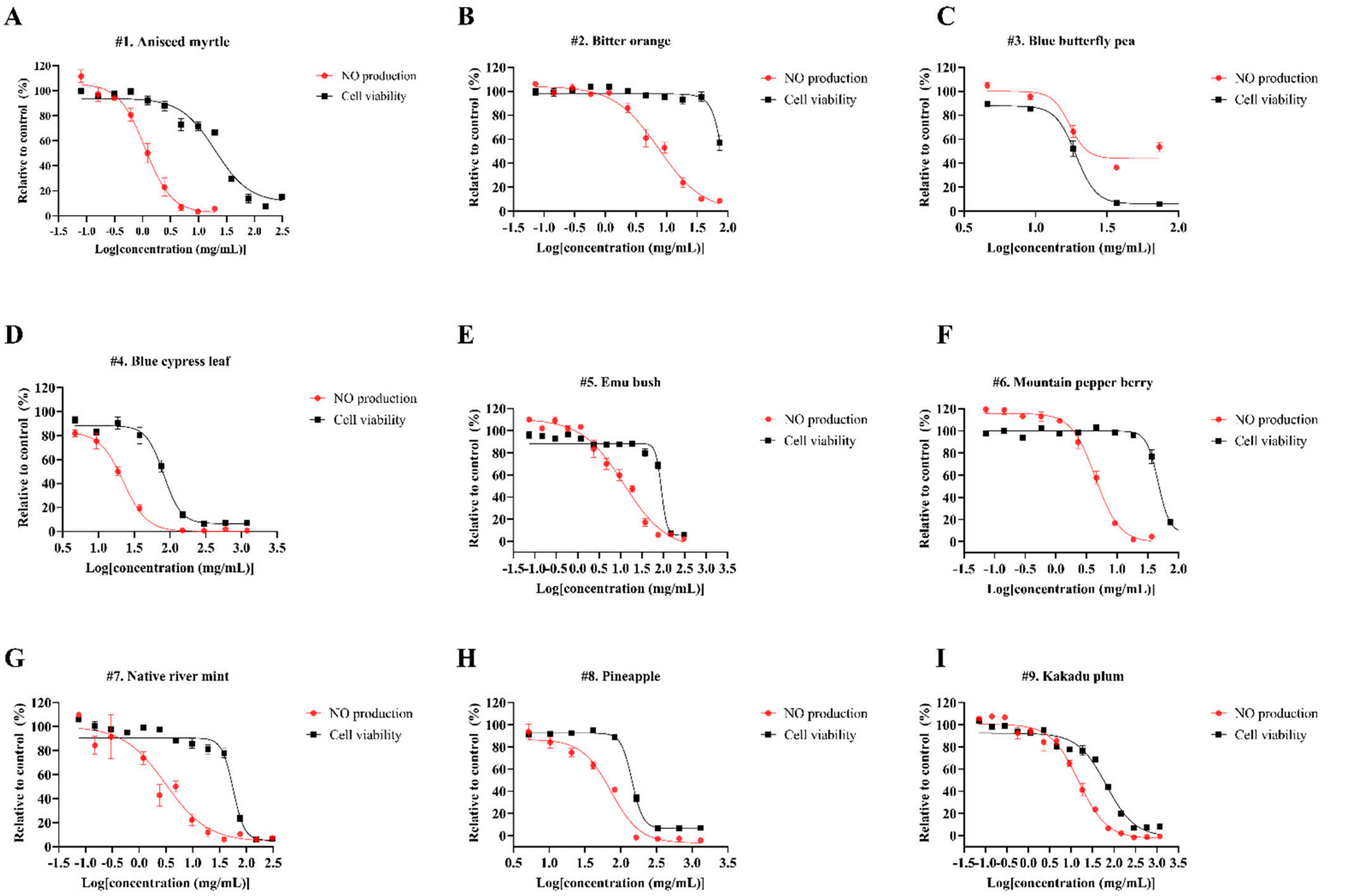

Figure S1. Dose-response curves of the individual NPE for NO inhibition and cell viability in LPS-induced RAW 264.7 (n=3 experiments). (A) #1. Aniseed myrtle (B) #2. Bitter orange (C) #3. Blue butterfly pea (D) #4. Blue cypress leaf (E) #5. Emu bush (F) #6. Mountain pepper berry (G) #7. Native river mint (H) #8. Pineapple and (I) #9. Kakadu plum. Figures were generated by GraphPad Prism 10.0. Results shown as mean ± SEM.

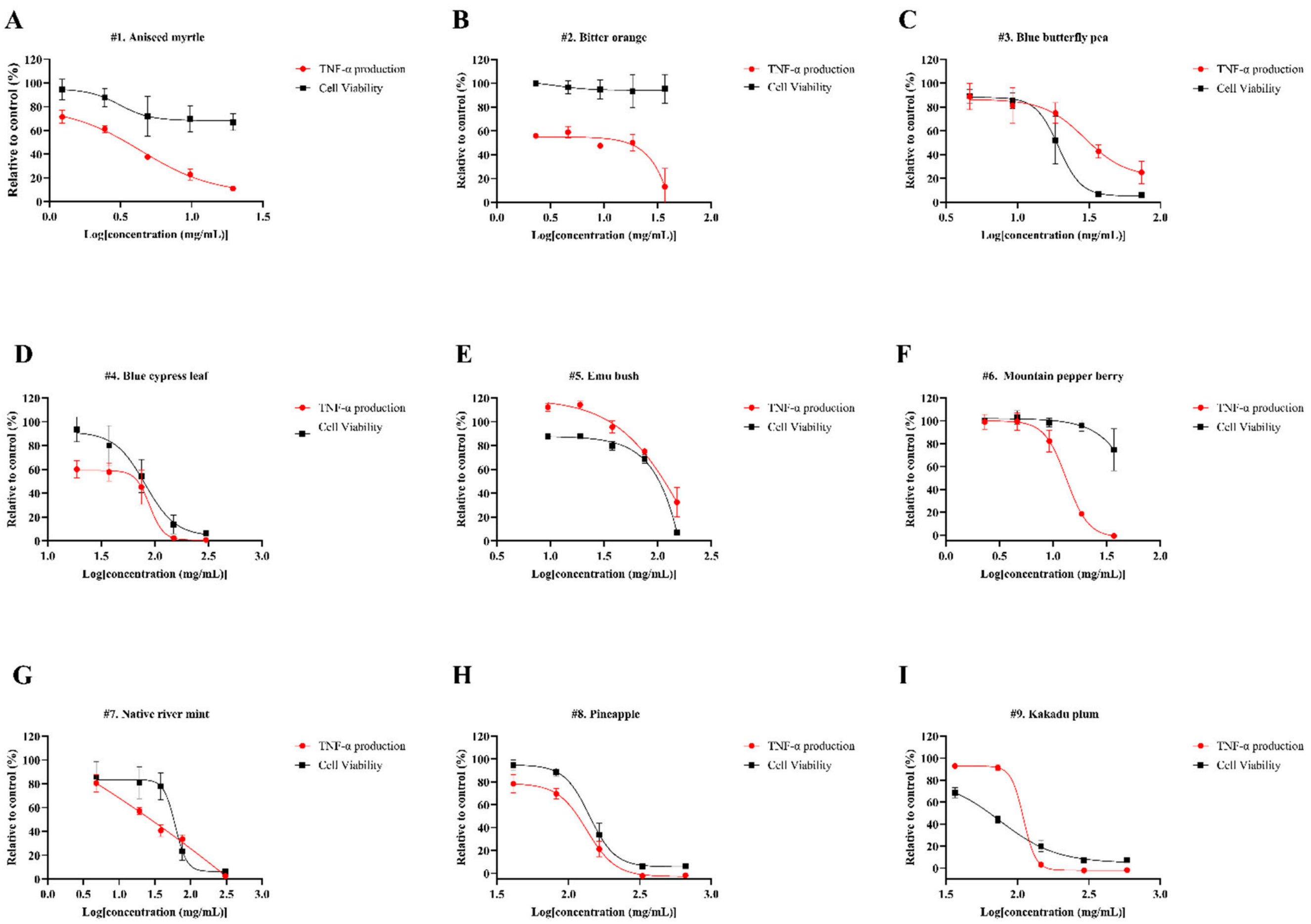

Figure S2. Dose-response curves of the individual NPE for TNF-α inhibition and cell viability in LPS-induced RAW 264.7 (n=3 experiments). (A) #1. Aniseed myrtle (B) #2. Bitter orange (C) #3. Blue butterfly pea (D) #4. Blue cypress leaf (E) #5. Emu bush (F) #6. Mountain pepper berry (G) #7. Native river mint (H) #8. Pineapple and (I) #9. Kakadu plum. Figures were generated by GraphPad Prism 10.0. Results shown as mean ± SEM.

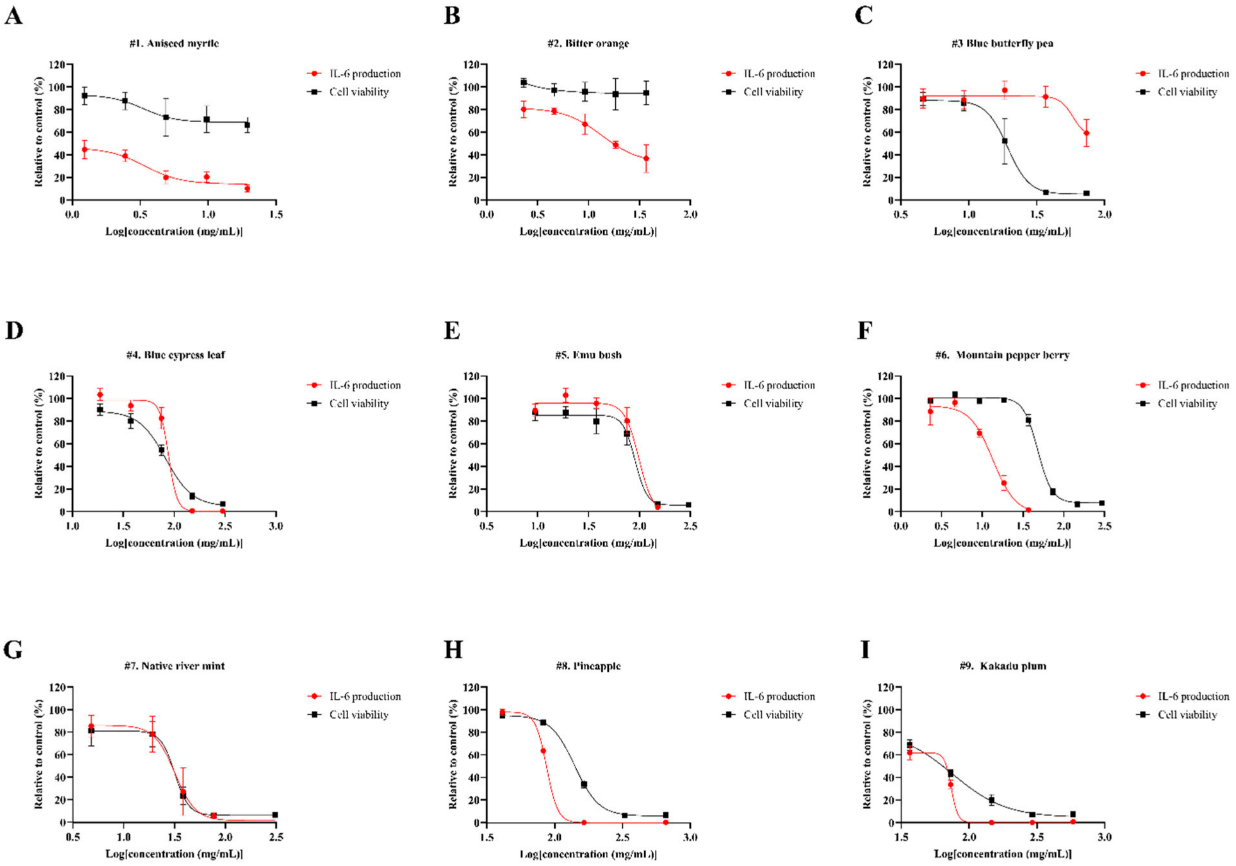

Figure S3. Dose-response curves of the individual NPE for IL-6 inhibition and cell viability in LPS-induced RAW 264.7 (n=3 experiments). (A) #1. Aniseed myrtle (B) #2. Bitter orange (C) #3. Blue butterfly pea (D) #4. Blue cypress leaf (E) #5. Emu bush (F) #6. Mountain pepper berry (G) #7. Native river mint (H) #8. Pineapple and (I) #9. Kakadu plum. Figures were generated by GraphPad Prism 10.0. Results shown as mean ± SEM.

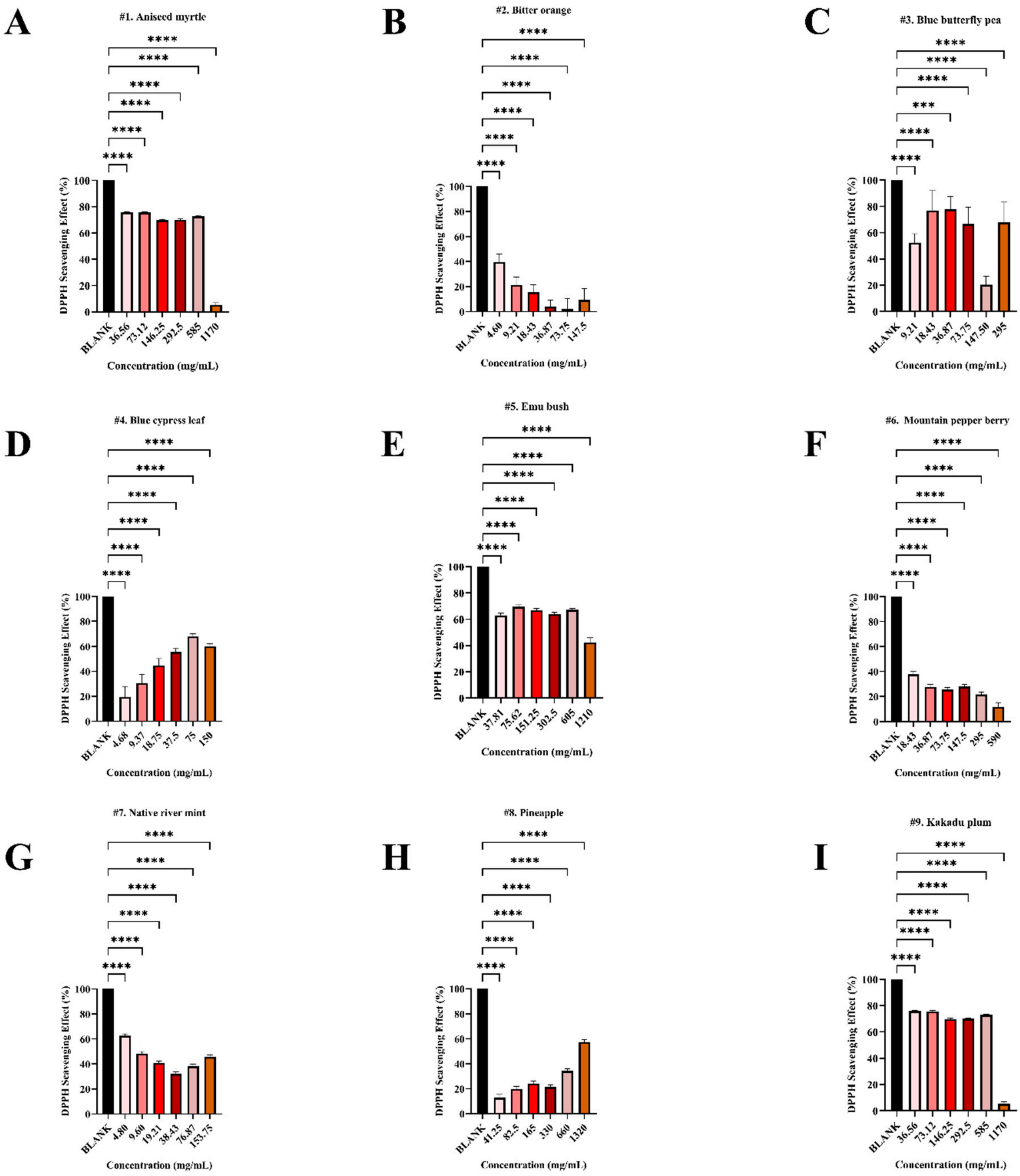

Figure S4. DPPH scavenging effect (%) for individual NPE (n=10 experiments). (A) #1. Aniseed myrtle (B) #2. Bitter orange (C) #3. Blue butterfly pea (D) #4. Blue cypress leaf (E) #5. Emu bush (F) #6. Mountain pepper berry (G) #7. Native river mint (H) #8. Pineapple and (I) #9. Kakadu plum. Figures were generated by GraphPad Prism 10.0. Results shown as mean ± SEM. A One-way ANOVA analysis was conducted to determine statistical significance among concentrations of extracts compared to blank (****p<0.0001 *vs* blank).

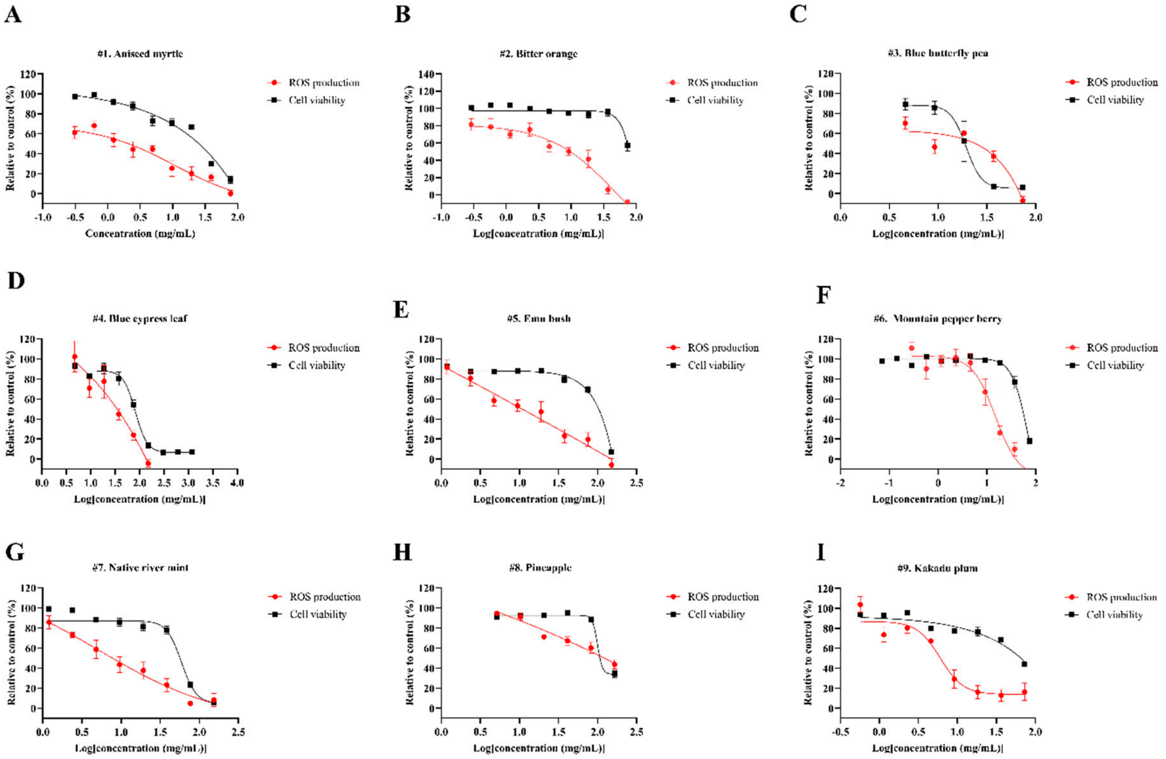

Figure S5. Dose-response curves of the individual NPE for ROS inhibition and cell viability in LPS-induced RAW 264.7 (n=3 experiments). (A) #1. Aniseed myrtle (B) #2. Bitter orange (C) #3. Blue butterfly pea (D) #4. Blue cypress leaf (E) #5. Emu bush (F) #6. Mountain pepper berry (G) #7. Native river mint (H) #8. Pineapple and (I) #9. Kakadu plum. Figures were generated by GraphPad Prism 10.0. Results shown as mean ± SEM.

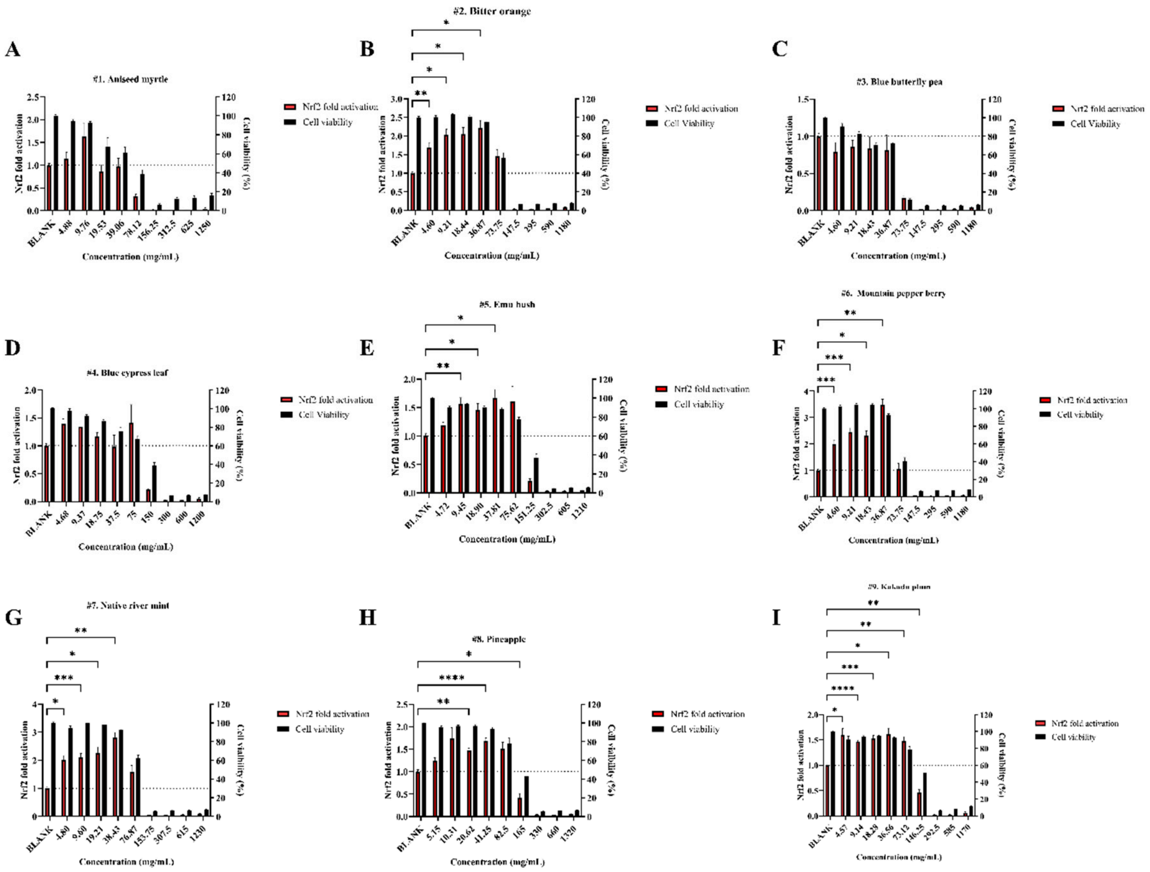

Figure S6. Nrf2 activation and cell viability of individual NPE on MCF7 AREc32 cells (n≥3 experiments). (A) #1. Aniseed myrtle (B) #2. Bitter orange (C) #3. Blue butterfly pea (D) #4. Blue cypress leaf (I) #5. Emu bush (F) #6. Mountain pepper berry (G) #7. Native river mint (H) #8. Pineapple (I) #9. Kakadu plum. Results shown as mean ± SEM. Figures were generated using GraphPad Prism 10.0. A One-way ANOVA analysis was conducted to determine statistical significance amongst concentration groups compared to blank. (**** p<0.0001, *** p<0.001, ** p<0.01 and * p<0.05 *vs* blank).

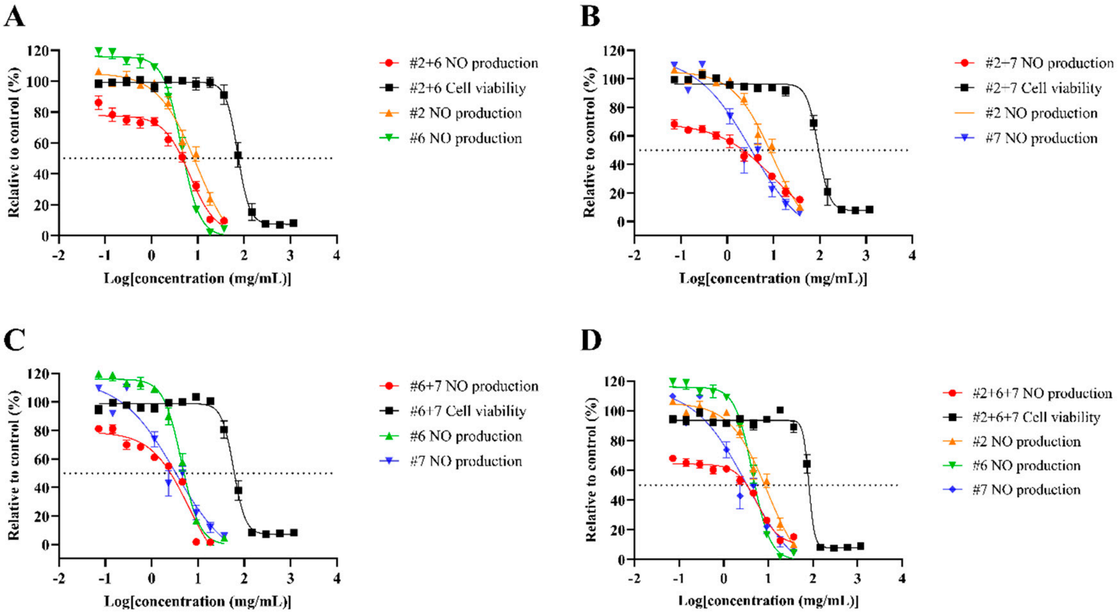

Figure S7. Dose response curves for NO inhibition and cell viability in LPS-induced RAW 264.7 cells of NPE combinations (1:1 or 1:1:1, *w*/*w*) of Bitter orange (#2), Mountain pepper berry (#6) and Native

river mint (#7) compared with their individual NO and cell viability (n≥3 experiments, mean ± SEM). (A) #2+6, (B) #2+7, (C) #6+7 and (D) #2+6+7. Figures were produced by GraphPad Prism 10.0.

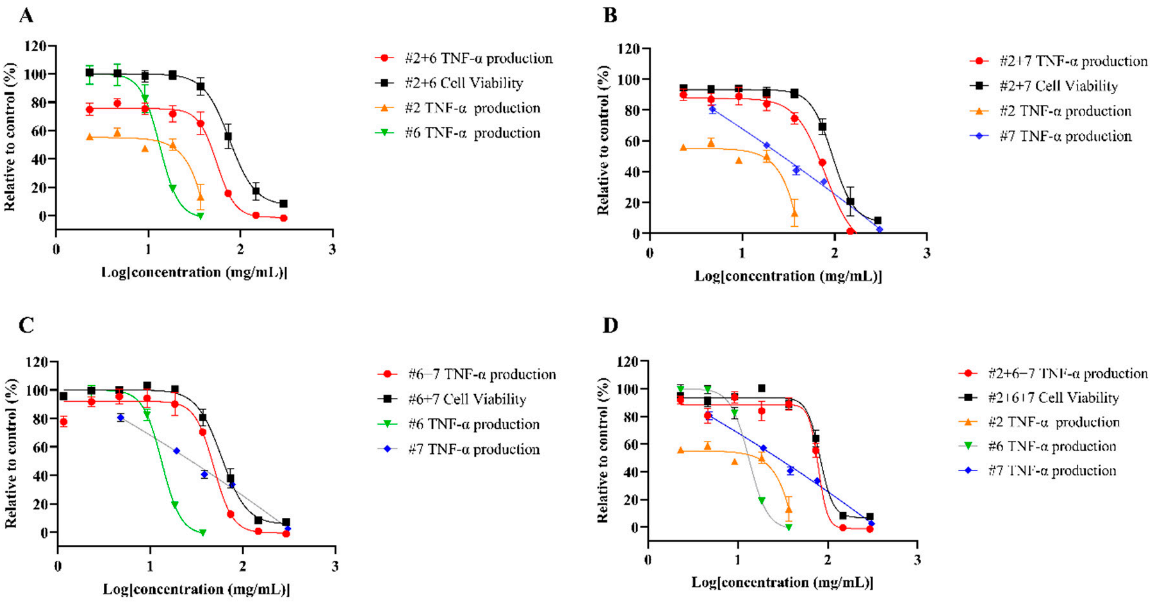

Figure S8. Dose response curves for TNF-α inhibition and cell viability in LPS-induced RAW 264.7 cells of NPE combinations (1:1 or 1:1:1, *w/w*) of Bitter orange (#2), Mountain pepper berry (#6) and Native river mint (#7) compared with their individual NO and cell viability (n≥3 experiments, mean ± SEM). (A) #2+6, (B) #2+7, (C) #6+7 and (D) #2+6+7. Figures were produced by GraphPad Prism 10.0.

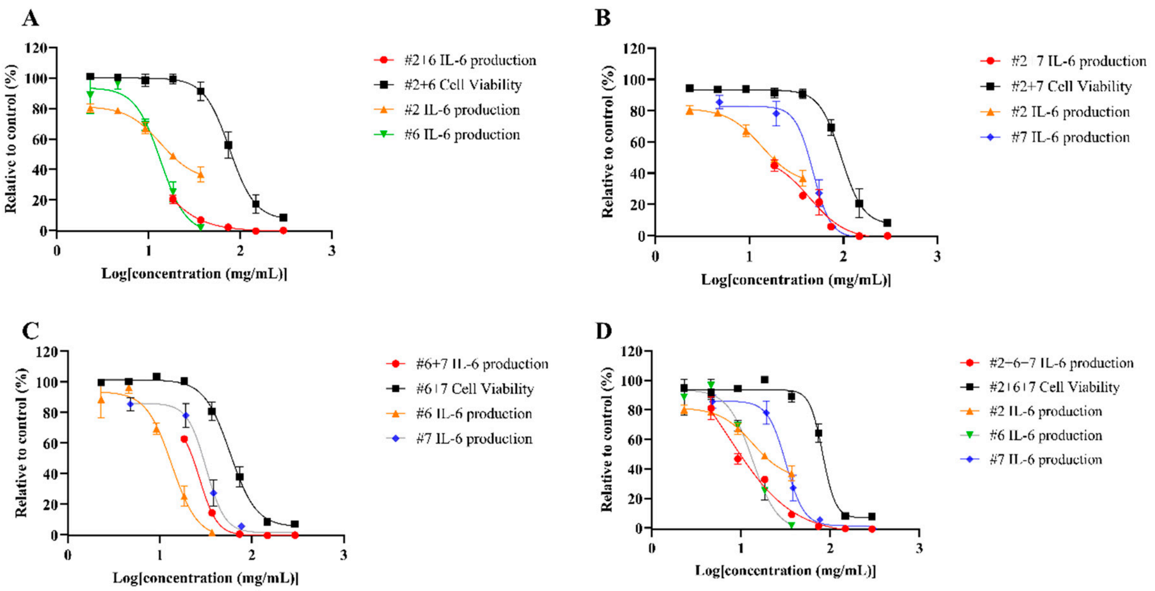

Figure S9. Dose response curves for IL-6 inhibition and cell viability in LPS-induced RAW 264.7 cells of NPE combinations (1:1 or 1:1:1, *w/w*) of Bitter orange (#2), Mountain pepper berry (#6) and Native river mint (#7) compared with their individual NO and cell viability (n≥3 experiments, mean ± SEM). (A) #2+6, (B) #2+7, (C) #6+7 and (D) #2+6+7. Figures were produced by GraphPad Prism 10.0.

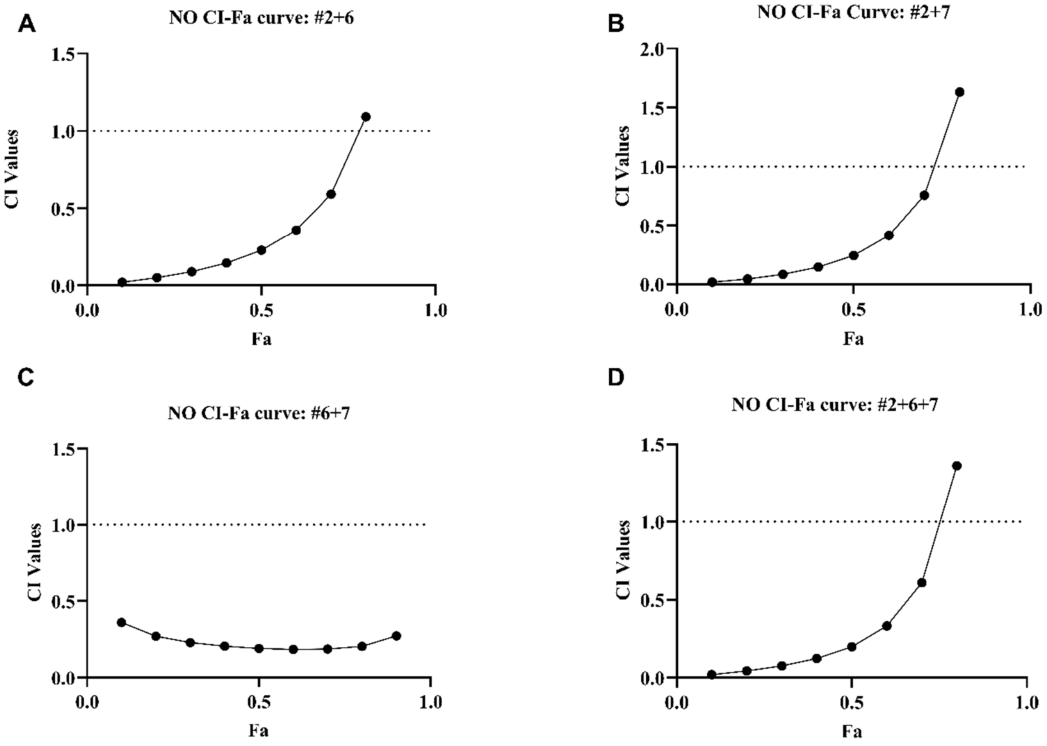

Figure S10. CI-Fa curves of NO inhibitory effect for NPE combinations (1:1 or 1:1:1, *w/w*) of Bitter orange (#2), Mountain pepper berry (#6) and Native river mint (#7). Figure includes CI-Fa curves for (A) #2+6, (B) #2+7, (C) #6+7 and (D) #2+6+7. Figures were generated by GraphPad Prism 10.0.

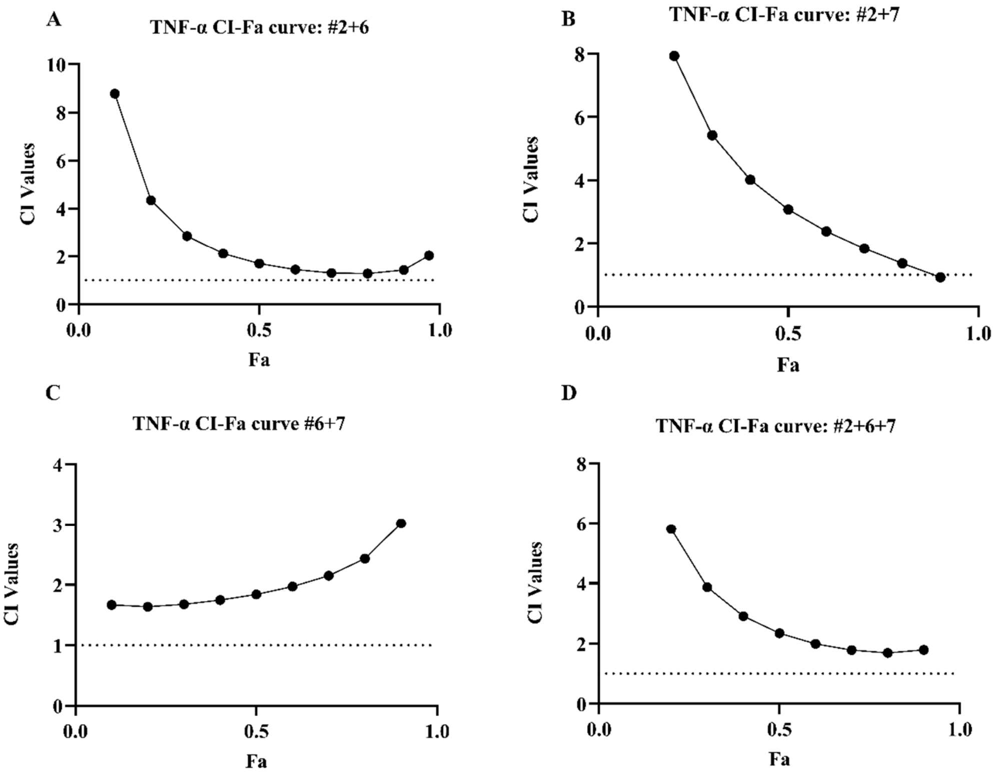

Figure S11. CI-Fa curves of TNF-α inhibitory effect for NPE combinations (1:1 or 1:1:1, *w/w*) of Bitter orange (#2), Mountain pepper berry (#6) and Native river mint (#7). Figure includes CI-Fa curves for (A) #2+6, (B) #2+7, (C) #6+7 and (D) #2+6+7. Figures were generated by GraphPad Prism 10.0.

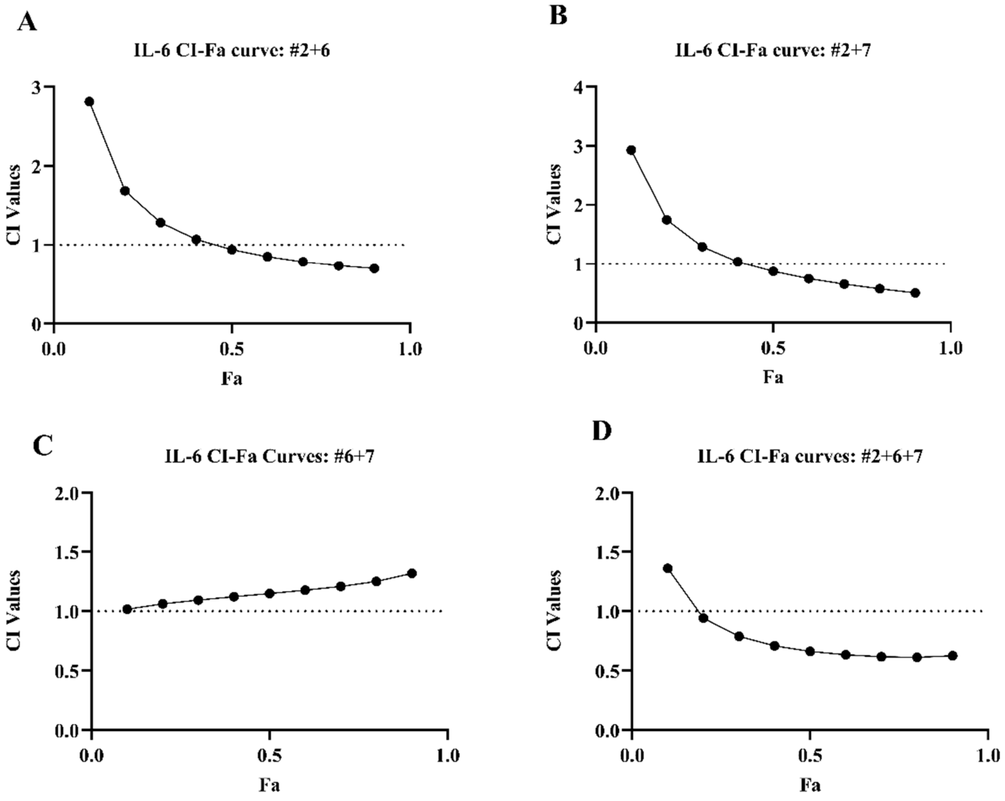

Figure S12. CI-Fa curves of IL-6 inhibitory effect for NPE combinations (1:1 or 1:1:1, *w/w*) of Bitter orange (#2), Mountain pepper berry (#6) and Native river mint (#7). Figure includes CI-Fa curves for (A) #2+6, (B) #2+7, (C) #6+7 and (D) #2+6+7. Figures were generated by GraphPad Prism 10.0.

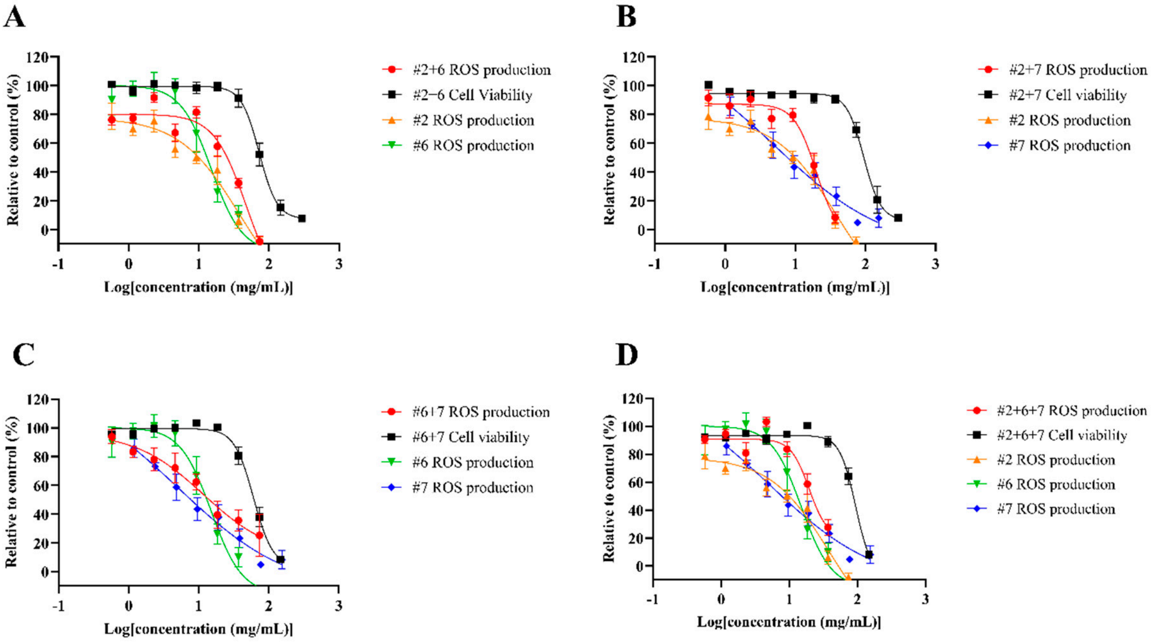

Figure S13. Dose response curves for ROS inhibition and cell viability in LPS-induced RAW 264.7 cells of NPE combinations (1:1 or 1:1:1, *w/w*) of Bitter orange (#2), Mountain pepper berry (#6) and Native river mint (#7) compared with their individual NO and cell viability (n≥3 experiments, mean ± SEM). (A) #2+6, (B) #2+7, (C) #6+7 and (D) #2+6+7. Figures were produced by GraphPad Prism 10.0.

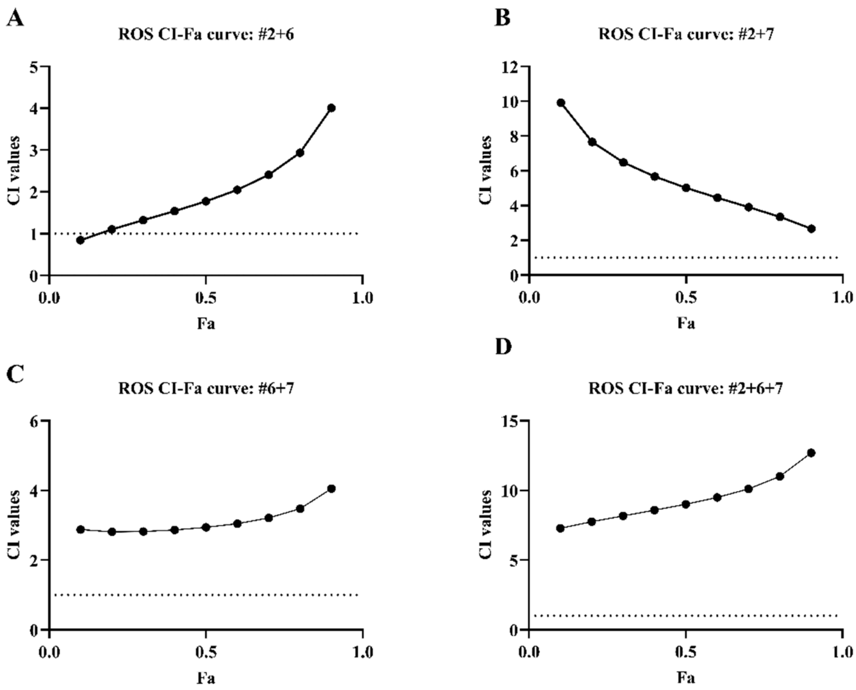

Figure S14. CI-Fa curves of ROS inhibitory effect for NPE combinations (1:1 or 1:1:1, *w/w*) of Bitter orange (#2), Mountain pepper berry (#6) and Native river mint (#7). Figure includes CI-Fa curves for (A) #2+6, (B) #2+7, (C) #6+7 and (D) #2+6+7. Figures were generated by GraphPad Prism 10.0.

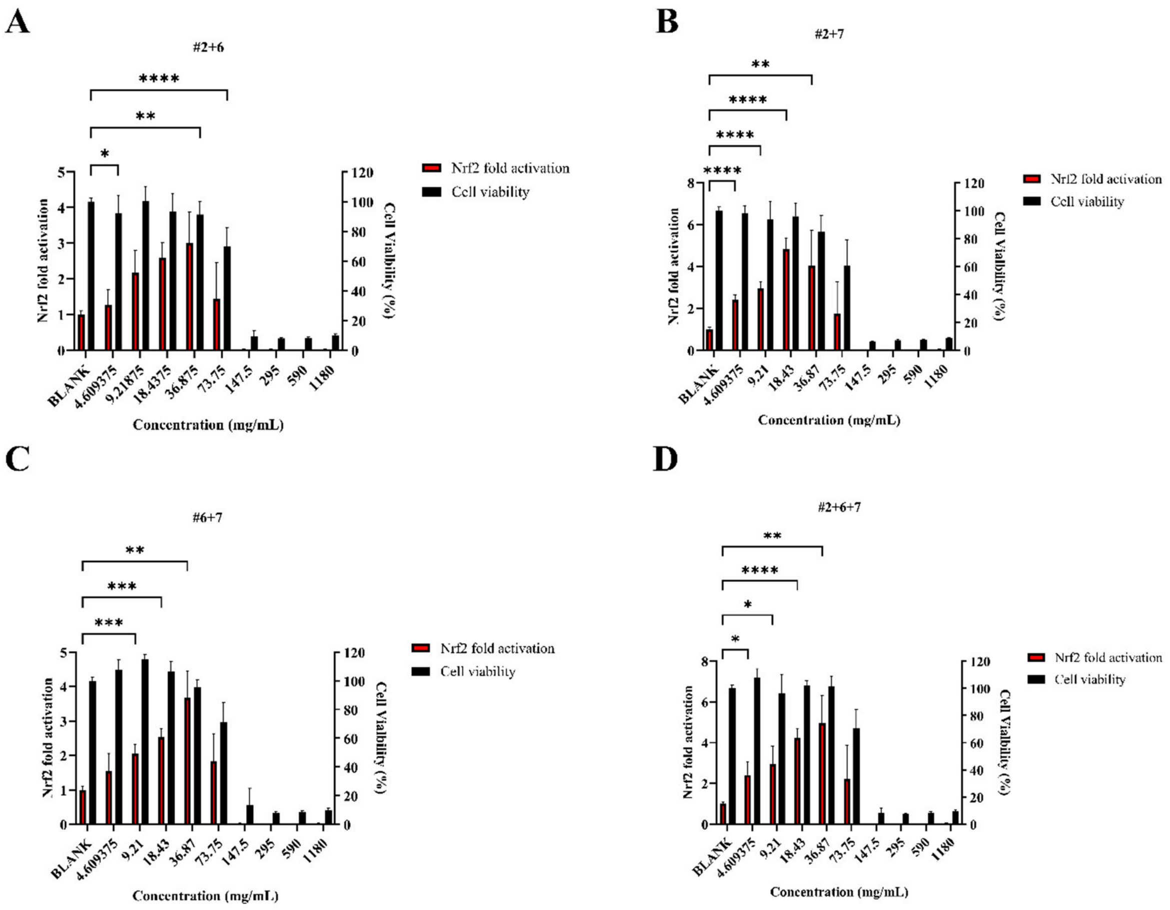

Figure S15. Nrf2 activation and cell viability of NPE combinations (1:1 or 1:1:1, *w/w*) of Bitter orange (#2), Mountain pepper berry (#6) and Native river mint (#7) extracts on MC7 AREc32 cells (n≥3

experiments) . (A) #2+6, (B) #2+7, (C) #6+7 and (D) #2+6+7. Results shown as mean ± SEM. Figures were generated using GraphPad Prism 10.0. A One-way ANOVA analysis was conducted to determine statistical significance amongst concentration groups compared to blank. (**** p<0.0001, *** p<0.001, ** p<0.01 and * p<0.05 *vs* blank).

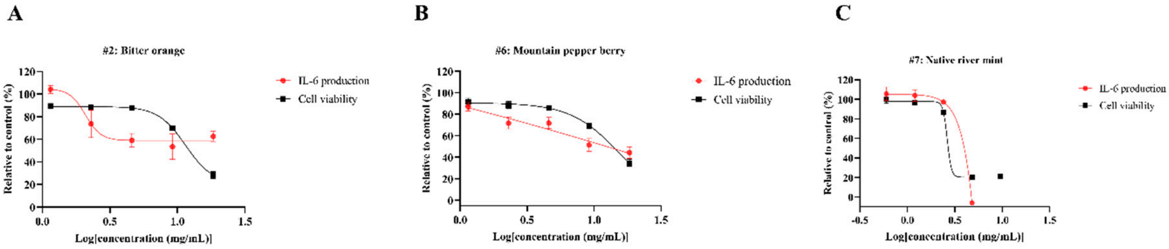

Figure S16. Dose response curves for IL-6 inhibition and cell viability in LPS-induced HDF cells (A) Bitter orange (#2), (B) Mountain pepper berry (#6) and (C) Native river mint (#7) compared with their individual NO and cell viability (n≥3 experiments, mean ± SEM). Figures were produced by GraphPad Prism 10.0.
